# Application of type-2 heptagonal fuzzy sets with multiple operators in multi-criteria decision-making for identifying risk factors of Zika virus

**DOI:** 10.1186/s12879-025-10741-9

**Published:** 2025-04-01

**Authors:** M. Sheela Rani, S. Dhanasekar

**Affiliations:** Department of Mathematics, School of Advanced Sciences, Vellore Institute of Technology, Chennai, 600127 Tamilnadu India

**Keywords:** Type-2 heptagonal fuzzy set, Type-2 heptagonal fuzzy number, MCDM, TOPSIS, VIKOR, WASPAS

## Abstract

**Purpose:**

This study aims to identify and rank the key risk factors associated with the Zika virus by leveraging a novel multi-criteria decision-making (MCDM) framework based on type-2 heptagonal fuzzy sets. By integrating advanced aggregation operators, the framework effectively addresses uncertainties in expert assessments and enhances decision-making reliability.

**Methods:**

A robust MCDM approach was developed using type-2 heptagonal fuzzy sets, which provide a more nuanced representation of uncertainty compared to traditional fuzzy models. These sets were selected due to their superior ability to handle vague, imprecise, and subjective expert judgments, common challenges in epidemiological risk assessments. Arithmetic and geometric aggregation operators were employed to process fuzzy data effectively. To ensure comprehensive and reliable rankings, the framework incorporated both outranking methods and distance-based approaches, specifically TOPSIS and WASPAS. A sensitivity analysis was conducted to validate the stability of the rankings under varying conditions.

**Results:**

The proposed framework identified $Z_3$ (unprotected sexual activity) as the most critical risk factor with a score of 0.6717, followed by $Z_8$ (blood transfusions) at 0.5783, $Z_{10}$ (pregnancy) at 0.5753, $Z_9$ (mosquito bites) at 0.4917, and $Z_7$ (travel to endemic areas) at 0.4726. The rankings remained consistent across different MCDM methods (TOPSIS and WASPAS), demonstrating the robustness of the proposed approach. Pearson correlation analysis confirmed a strong agreement between methods, with correlation coefficients, reinforcing the reliability of the model.

**Conclusion:**

This study introduces an advanced decision-support system for healthcare professionals to systematically identify and prioritize Zika virus risk factors. By leveraging type-2 heptagonal fuzzy sets, the framework effectively captures and processes uncertainty stemming from incomplete epidemiological data, imprecise expert assessments, and subjective linguistic evaluations. The consistency of rankings across multiple MCDM methods, along with sensitivity analysis confirming their stability, demonstrates the model’s reliability. These findings provide a scientifically grounded tool for improving risk analysis and strategic public health interventions.

## Introduction

Zika virus is an arthropod-borne virus (arbovirus) transmitted primarily by Aedes mosquitoes, particularly *Aedes aegypti* and *Aedes albopictus*. It belongs to the Flavivirus genus, which includes other significant pathogens like dengue, west Nile, and yellow fever viruses. Zika virus was first isolated from a rhesus monkey in the Zika Forest of Uganda in 1947. The virus was later identified in Aedes mosquitoes in the same region [1]. The first human cases of Zika virus infection were reported in Uganda and the United Republic of Tanzania in the early 1950s. Serological studies confirmed human exposure to the virus in subsequent decades across various regions of Africa and Asia [2].

The virus gained international attention during outbreaks in the Pacific Islands, particularly in Yap State, Federated States of Micronesia, in 2007. This event marked the first major outbreak outside of Africa and Asia [3]. During the outbreak in Brazil, an increase in cases of microcephaly and other neurological disorders in newborns was reported, leading to heightened concern and research into the virus’s potential teratogenic effects [4]. In 2016, the WHO declared the Zika virus a PHEIC due to its rapid spread and potential health impacts, especially in pregnant women and newborns [5, 6].

The first cases of Zika virus in India were detected in Ahmedabad, Gujarat. The Ministry of Health and Family Welfare confirmed three cases through laboratory testing conducted by the NIV, Pune. In 2018, an outbreak occurred in Jaipur, Rajasthan, with over 150 reported cases [7]. The outbreak prompted extensive vector control measures and public health interventions. Continuously in 2019, additional cases were reported in Bhopal, Madhya Pradesh, indicating the virus’s ability to spread to new regions. The Indian government initiated enhanced surveillance, public awareness campaigns, and guidelines for clinicians on managing Zika virus infections.

Zika virus was first identified in India in 1952, but it remained largely undetected until recent years. The ICMR has confirmed [8] the presence of the virus in several states, including Uttar Pradesh, Kerala, Telangana, Jharkhand, Rajasthan, Punjab, and Delhi. Enhanced surveillance in 2021 highlighted the silent spread of the Zika virus across India, with local transmission confirmed in these states. The re-emergence and spread of Zika in India underline the importance of continuous monitoring and research.

Munivenkatappa et al. [9] reported the initial human case of Zika virus disease in Karnataka, subsequent to the virus’s detection in Aedes aegypti mosquitoes. This finding underscores the importance of vigilant vector surveillance and public health preparedness in the region.

In 2024, India reported a total of 151 Zika virus disease cases across three states: Maharashtra (140 cases), Karnataka (10 cases), and Gujarat (1 case). Notably, the Pune district in Maharashtra accounted for 125 of these cases, highlighting it as a significant hotspot.

India [10] Developing effective vaccines and treatments is critical. Bharat Biotech’s ongoing development of a Zika virus vaccine, which is entering phase two clinical trials, represents a significant step toward providing a preventive solution for the population. Strengthening surveillance systems for the Zika virus, along with dengue and chikungunya, can enhance early detection and response, minimizing outbreaks. Effective monitoring can also help understand the spectrum of the disease in newborns and the general population. Public awareness campaigns about the prevention of mosquito bites and transmission methods are vital. Preventive measures, such as controlling mosquito breeding sites and using protective measures, are essential in reducing the risk of Zika virus transmission. Kumar et al. [11] highlights the increasing prevalence of CZS in India and emphasizes the need for comprehensive public health strategies to address this issue. The authors discuss the multifaceted challenges posed by the Zika virus, particularly its impact on newborns, and call for enhanced surveillance, preventive measures, and healthcare interventions to mitigate the effects of CZS in the country.

Waafira et al. [12] provided a comprehensive overview of the increasing incidence of Zika virus infections in India. It details the clinical manifestations, and potential complications, and emphasizes preventive strategies to curb the spread of the virus.

**Fig. 1:**
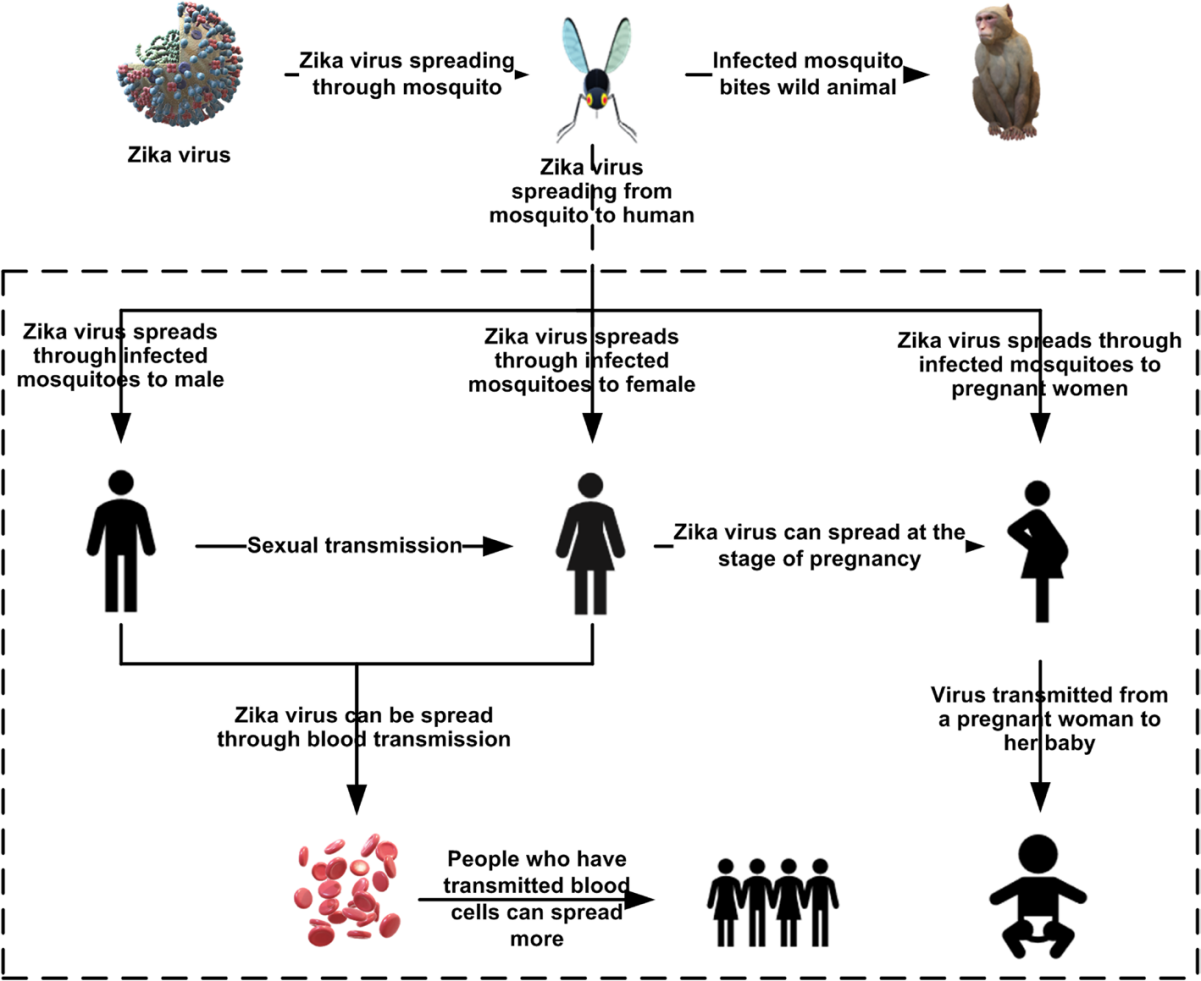
Zika virus transmission routes

Figure 1 shows the transmission routes of Zika virus in human beings.

Through this, Research and proactive measures are essential to manage and mitigate the impact of the Zika virus in India. Continued surveillance, development of vaccines, and public health initiatives will play a pivotal role in addressing this emerging threat. The collaboration between public health authorities, researchers, and the community is crucial to control the spread and prevent the severe consequences of Zika virus infections.

### Fuzzy set theory

Fuzzy set theory, introduced by Lotfi A. Zadeh [13] in 1965, represents a significant departure from classical set theory by allowing elements to have degrees of membership. Unlike classical binary sets, where an element either belongs or does not belong to a set, fuzzy sets accommodate partial membership, ranging from [0, 1].

IFS introduced by Atanassov [14], these sets consider both membership and non-membership degrees, providing a richer framework for handling uncertainty. HFS [15] allows for multiple membership values for an element, reflecting hesitation and uncertainty in the membership assignment. IVFS [16] uses intervals to represent the membership degrees, providing a straightforward way to capture uncertainty. Different types of fuzzy sets are constructed by different authors for various applications. Such as, TFS [17], TrFS [18], PFS [19], T2PFS [20], CPFS [21], OFS [22], Q-ROFS [23], PeFS [24], HFS [25].

T2FS, first introduced by Zadeh in 1975, extends traditional fuzzy sets by allowing membership values to be fuzzy as well. This extension enables a more refined representation of uncertainty, providing a flexible tool for handling data with varying degrees of vagueness and imprecision. T2FS incorporate two levels of membership: a primary membership function and a secondary membership function, offering a more comprehensive way to model uncertainty. The field continued to evolve with the development of new types of fuzzy sets, such as Type-2 triangular fuzzy sets, Type-2 trapezoidal fuzzy sets, and Type-2 pentagonal fuzzy sets, etc., each addressing different aspects of uncertainty and imprecision.

This work introduces T2HFS for addressing the multi-dimensional nature of uncertainty present in many real-world applications, particularly in public health decision-making, where expert opinions often vary and the available data is incomplete or imprecise. Their ability to represent a broader spectrum of membership values allows decision-makers to better capture complex, subjective, and imprecise information. This makes them particularly valuable in MCDM frameworks, as they offer a more accurate representation of the decision environment compared to traditional methods.

The origin of T2HFS lies in the need to model more precise and multi-dimensional uncertainty. While traditional fuzzy sets and even simpler T2FS can effectively handle many situations, T2HFS introduces a higher degree of granularity, allowing for a better fit when dealing with highly imprecise data.

T2HFS represents an advanced variation of T2FS that is designed to provide more nuanced and flexible modeling in decision-making frameworks. These fuzzy sets are characterized by seven vertices, allowing for seven distinct degrees of membership and a more complex, yet accurate, representation of uncertainty. They are particularly suited for scenarios where multiple sources of ambiguity exist, making them highly effective in complex decision-making environments.

Thus, Type-2 fuzzy set theory became integral to numerous applications, from consumer electronics to medical diagnosis.

### MCDM methods

MCDM methods are a set of techniques used to evaluate and prioritize multiple competing criteria in decision-making processes. Originating in the 1960s with the development of linear programming and decision theory, MCDM methods have evolved to address complex decision problems in various fields. Key methods include the AHP, introduced by Thomas Saaty [26] in the 1970s, and ANP [27] in 1996. These methods provide structured frameworks for decision-makers to assess alternatives based on multiple, often conflicting criteria.

MCDM methods are applied across various fields, providing a systematic approach to ranking and prioritizing alternatives based on multiple criteria. They help in decision-making by evaluating options in diverse domains such as healthcare, finance, supply chain management, and environmental planning. Kumar and Pamucar [28] provides a comprehensive review of MCDM methods from 2004 to 2024, offering a systematic comparison of their applications, advantages, and limitations. It serves as a valuable resource for researchers and practitioners in selecting appropriate MCDM techniques for complex decision-making problems.

Relevant literature has been provided to support this such as, [29] presents a vendor selection model using the AHP combined with fuzzy preference programming. It addresses the uncertainty in vendor evaluations and decision-making by incorporating fuzzy logic into the AHP framework. The study aims to improve the accuracy and reliability of selecting optimal vendors for organizational needs.

Dutta et al. [30] compares the TOPSIS and TODIM methods for evaluating the performance of foreign players in the Indian premier league. It analyzes multiple criteria to rank players based on their contributions, highlighting the strengths and differences of both MCDM approaches. The results provide insights into decision-making in sports analytics, emphasizing the effectiveness of these methods in ranking player performance. Kaur and Priya [31] discusses the selection of an optimal inventory policy within a Pythagorean fuzzy environment. It explores how the PFS theory can be applied to inventory management, focusing on decision-making under uncertainty. The study provides insights into the use of fuzzy logic for enhancing the efficiency of inventory systems.

Gul [32] enhances the VIKOR approach by integrating bipolar fuzzy preference $\delta$ -covering and bipolar fuzzy rough sets, improving decision-making under uncertainty. It provides a more refined preference structure, ensuring better accuracy in MCDM problems [33] enhances occupational risk analysis by integrating fuzzy logic with the Fine-Kinney model, providing a more precise and adaptable decision-support system for risk prioritization under uncertainty. Bouraima et al. [34] applies the AROMAN MCDM approach to optimize sustainable healthcare system devolution strategies, ensuring efficient resource allocation and improved decision-making in healthcare management.

MCDM methods are significantly impactful in medical diagnosis and have broader applications across various fields due to their ability to handle complex decision scenarios involving multiple criteria. MCDM methods allow healthcare professionals to integrate and prioritize diverse diagnostic criteria such as symptoms, test results, patient history, and risk factors. This holistic approach enhances diagnostic accuracy by considering a comprehensive set of factors. These methods incorporate patient preferences and values, facilitating personalized medicine. By weighing criteria based on patient-specific factors, MCDM enhances treatment plans that align with individual patient needs. It provides structured frameworks for clinical decision support systems. They assist healthcare providers in making informed decisions by quantifying uncertainties and providing systematic evaluations.

Hence, MCDM methods play a crucial role in enhancing decision-making processes across medical diagnosis and various other applications, contributing to improved outcomes, efficiency, and informed decision-making in complex decision scenarios.

### Literature review on TOPSIS, VIKOR and WASPAS

TOPSIS ranks alternatives based on their proximity to an ideal solution and furthest from the negative ideal solution, providing a straightforward ranking mechanism useful in scenarios where clear distinctions between ideal and non-ideal solutions are crucial [35]. Recent articles such as [36] address the critical issue of flood risk assessment in coastal cities. By combining the EWM-TOPSIS method with machine learning techniques, the study offers an innovative approach to enhance the accuracy and reliability of flood risk evaluations. This research contributes to advancing methodologies for disaster risk reduction, particularly in vulnerable coastal urban areas. Indelicato et al. [37] explores the impact of attitudes towards homeworking on travel demand. By employing a fuzzy-hybrid TOPSIS method, the study offers insights into optimizing commuting patterns and urban mobility strategies, contributing to sustainable urban planning and policy-making. Chatterjee and Chakraborty [38] examines how different objective weighting methods influence the parametric optimization of non-traditional machining processes using the TOPSIS approach. This research is pivotal for industrial sectors aiming to enhance decision-making accuracy in optimizing manufacturing processes.

VIKOR emphasizes compromise solutions, balancing conflicting criteria to identify alternatives that best meet multiple objectives simultaneously [39]. Its ability to handle complex trade-offs makes it suitable for decision-making in environments with diverse and sometimes conflicting goals. Recent work [40] introduces an innovative decision-making framework combining Fermatean fuzzy sets with the best worst method and VIKOR methods, enhancing precision in selecting optimal waste treatment technologies. It addresses complex criteria such as environmental impact, cost-effectiveness, and regulatory compliance, crucial for sustainable healthcare practices. The research contributes to improving waste management efficiency, reducing environmental risks, and advancing sustainable healthcare infrastructure planning and implementation. Dağıstanlı [41] introduces a novel approach using interval-valued intuitionistic fuzzy sets with the VIKOR method, improving the robustness of R&D project selection. This methodology addresses uncertainty and subjective evaluations inherent in defense investments, enhancing decision accuracy and strategic planning. The research contributes to optimizing resource allocation, maximizing project success rates, and bolstering technological innovation in defense sectors globally. Kansal and Kumar [42] introduces an enhanced VIKOR method integrating intuitionistic fuzzy exponential knowledge and similarity measures, enhancing accuracy in complex decision scenarios. This approach accommodates uncertainties and diverse criteria, making it valuable for applications in diverse fields like business management, engineering, and environmental assessment, where precise and comprehensive decision support is crucial.

WASPAS combines the strengths of weighted sum and weighted product models, offering flexibility in aggregating criteria weights and performance scores. It accommodates varying degrees of importance assigned to different criteria, enhancing decision-making robustness [43]. Some recent works such as [44] introduce a strategic framework combining q-rung picture fuzzy AHP with WASPAS for the green supply chain management. This innovative model addresses complexities in decision-making by integrating fuzzy logic and multi-criteria evaluation, enhancing precision in selecting environmentally friendly supply chain strategies. The research contributes to reducing carbon footprints, optimizing resource usage, and promoting sustainable development, pivotal for mitigating environmental impacts in the energy industry. Dwivedi and Sharma [45] employs entropy and WASPAS techniques to evaluate suitable renewable energy resources, addressing the nation’s energy security and sustainability challenges. This research aids in optimizing resource allocation, minimizing environmental impact, and promoting energy independence through informed policy decisions. The findings are crucial for policymakers, energy planners, and stakeholders in advancing renewable energy adoption and achieving India’s climate goals effectively.

The hybrid MCDM methodology [46] enhances decision-making precision by integrating fuzzy logic, allowing for comprehensive risk assessment and mitigation strategies. The research supports healthcare providers in improving patient safety, optimizing resource allocation, and advancing medical decision support systems, thereby contributing to enhanced healthcare outcomes and operational efficiency in organ transplantation procedures. Sheela and Dhanasekar [47] introduces type-2 inter-valued trapezoidal fuzzy numbers with the operations of Einstein aggregation terms with TOPSIS and VIKOR algorithms with weighted AHP method. Similarly, [48] enhances the accuracy and reliability of evaluating service quality, crucial for ensuring user satisfaction, compliance with medical standards, and effective healthcare delivery through mobile applications. The research contributes to advancing healthcare technology by providing robust frameworks for evaluating and improving the performance of mobile medical apps, benefiting both users and healthcare providers alike. Demir and Moslem [49] introduces an innovative fuzzy MCDM approach tailored for optimizing the management of medical waste, which has become increasingly critical during public health emergencies. This methodology aids in prioritizing disposal methods, ensuring safety protocols, and minimizing environmental impact. The research addresses urgent challenges in healthcare infrastructure resilience, offering practical solutions to enhance pandemic response capabilities and sustainability in medical waste management. Its findings are instrumental for policymakers, healthcare professionals, and waste management authorities in mitigating health risks and promoting sustainable practices during global health crises.

Sheela Rani and Dhanasekar [50] aids in identifying critical factors influencing disease severity, transmission dynamics, and public health interventions. By integrating fuzzy logic, the study enhances decision support systems for healthcare authorities, contributing to improved pandemic preparedness, response strategies, and mitigation efforts. Its findings provide valuable insights into understanding and managing infectious disease risks effectively during global health crises. Ramdani et al. [51] addresses the critical issue of energy efficiency in industrial operations, a key component of sustainable development. The comparative analysis of TOPSIS and VIKOR provides valuable insights for researchers and practitioners seeking to implement energy optimization strategies in drilling and other manufacturing processes.

### Motivation

 

- **Complexity of Zika Virus Risk Factors:** The Zika virus presents multifaceted risk factors that demand sophisticated analytical approaches. Type-2 heptagonal fuzzy numbers provide a robust framework to capture the inherent uncertainties in the assessment of these risk factors.
- **Enhanced Precision with Type-2 Heptagonal Fuzzy Numbers:** Traditional fuzzy numbers might fall short in dealing with the ambiguity and vagueness associated with Zika virus risk factors. Type-2 heptagonal fuzzy numbers enhance the precision of modeling by accommodating higher levels of uncertainty.
- **TOPSIS Method for Effective Decision Making:** The TOPSIS method is known for its effectiveness in ranking and selecting among complex alternatives. Applying TOPSIS to type-2 heptagonal fuzzy numbers helps in systematically identifying and prioritizing Zika virus risk factors.
- **VIKOR Method for Compromise Solutions:** The VIKOR method focuses on providing compromise solutions for multi-criteria decision-making problems. By integrating VIKOR with type-2 heptagonal fuzzy numbers, it is possible to identify Zika virus risk factors that represent a balanced trade-off among conflicting criteria.
- **WASPAS Method for Weighted Aggregation:** The WASPAS method combines the advantages of the WSM and WPM. Utilizing WASPAS with type-2 heptagonal fuzzy numbers enhances the aggregation and evaluation of diverse risk factors associated with the Zika virus.
- **Application in Public Health:** The application of type-2 heptagonal fuzzy numbers, along with TOPSIS, VIKOR, and WASPAS methods, provides a comprehensive and systematic approach to identifying and prioritizing Zika virus risk factors. This can aid public health authorities in devising targeted strategies and interventions to mitigate the impact of the virus.

At last, Identifying and prioritizing the risk factors of the Zika virus is crucial due to its severe health implications. Advanced methodologies, including T2HFN and MCDM techniques like TOPSIS, VIKOR, and WASPAS, offer a comprehensive approach to tackling this challenge.

**Fig. 2:**
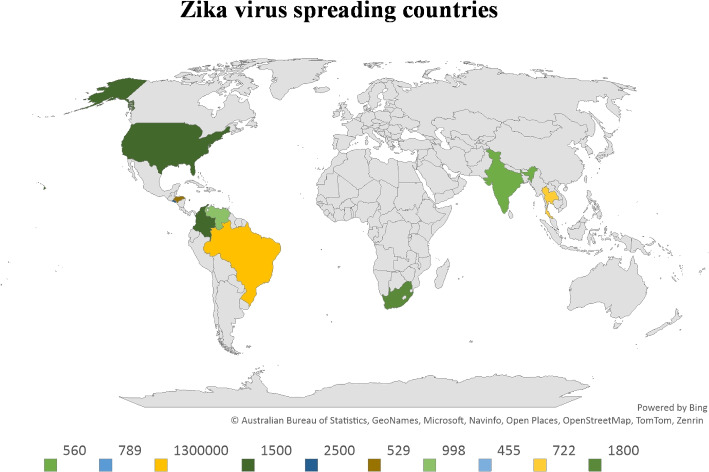
Map representation of the Zika virus spread

**Fig. 3:**
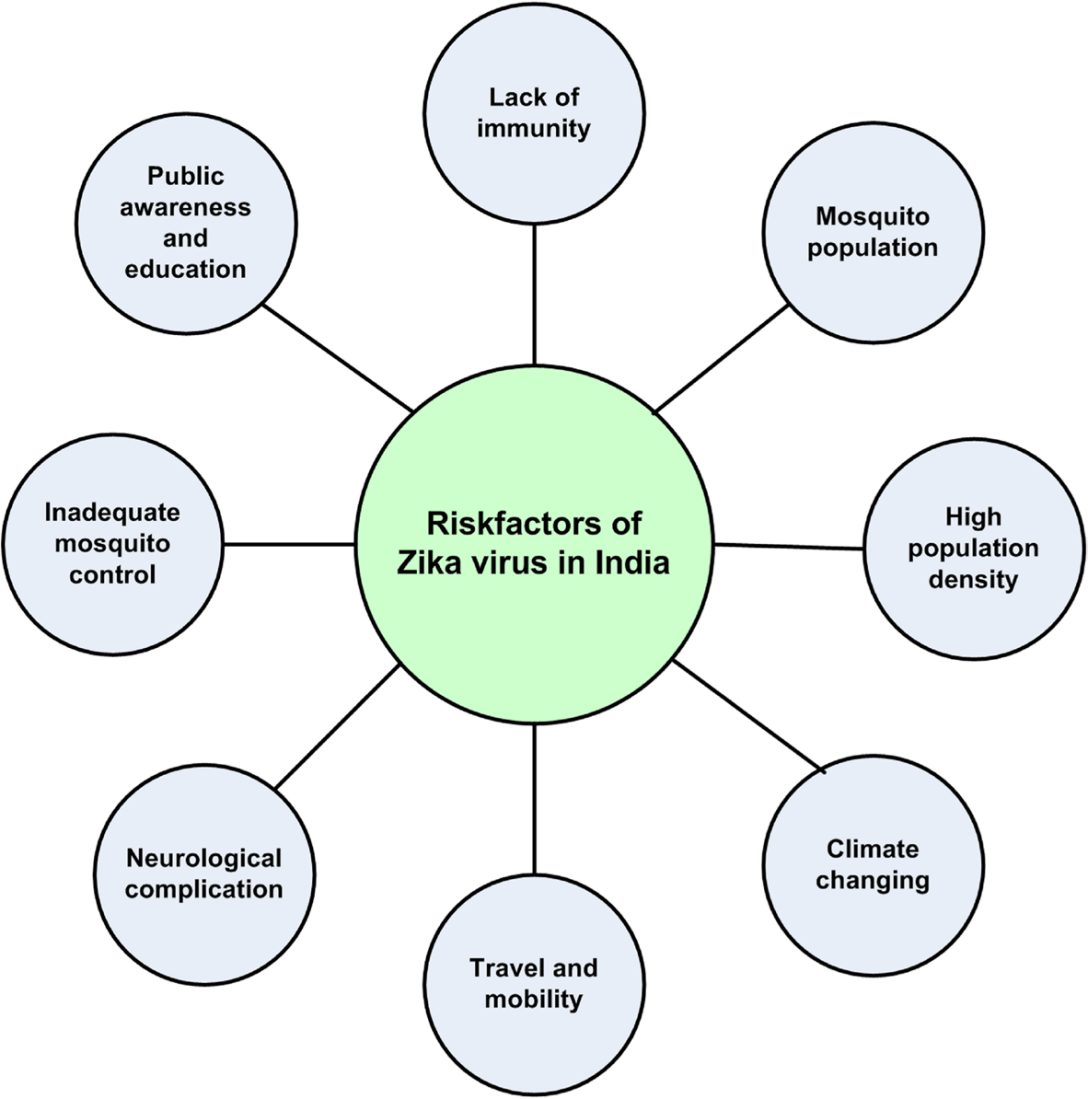
Risk factors of Zika virus in India

Jin et al. [52] provided the Zika virus spreading the countries and the Fig. 2 represented the spreading situation of Zika virus in all over the world. Figure 3 shows the main risk factors of the Zika virus in India.

### Contributions and novelties

1. This work pioneers the application of type-2 heptagonal fuzzy numbers in the field of epidemiology, providing a novel approach to managing the uncertainty and imprecision in assessing Zika virus risk factors.
2. The combination of type-2 heptagonal fuzzy numbers with these decision-making techniques establishes a comprehensive framework that can be applied to various public health challenges beyond the Zika virus.
3. The use of type-2 fuzzy logic improves the precision of risk factor analysis, leading to more informed and effective public health interventions.
4. The integration of TOPSIS, VIKOR, and WASPAS methods ensures a balanced and weighted evaluation of risk factors, accommodating trade-offs and aggregated assessments that traditional methods overlook.
5. This study introduces algorithms for type-2 heptagonal fuzzy TOPSIS, VIKOR, and WASPAS, employing a scoring mechanism to convert type-2 heptagonal fuzzy numbers into crisp values.
6. The proposed approach offers practical tools for public health authorities to systematically identify, prioritize, and address the most critical risk factors associated with the Zika virus, facilitating targeted and efficient response strategies.

### Structure

In this work, “Preliminaries” section explains the basic concepts of fuzzy sets and the definition of T2HFS, and T2HFNs with score function, weighted score function, accuracy function, t-norm and t-conorm, and set operations of T2HFS and T2HFNs. “Type-2 heptagonal fuzzy arithmetic aggregation operator” section explains T2HFWAO, T2HFOWAO with examples. “Type-2 heptagonal fuzzy geometric aggregation operators” section provides T2HFWGO, T2HFOWGO with examples. “Type-2 heptagonal fuzzy power arithmetic operator” section shows T2HFPWAO, T2HFPWOAO with examples. “Type-2 fuzzy heptagonal numbers extended to TOPSIS, VIKOR and WASPAS” section expounds the algorithm of the common structure of MADM, TOPSIS, VIKOR, and WASPAS. It explains the risk factors of the Zika virus and the calculation parts of this work. “Discussion” section contains an explanation of rankings, sensitivity analysis, limitations, and future work directions. For the abbreviations and their meanings used in this work, please refer to Table 1.

**Table 1:** Abbreviations and their means

Abbreviations	Means
MCDM	Multi-criteria decision-making
TOPSIS	Technique for Order of Preference by Similarity to Ideal Solution
VIKOR	VlseKriterijumska Optimizacija I Kompromisno Resenje
AHP	Analytic Hierarchy Process
BWM	Best Worst Method
WASPAS	Weighted Aggregated Sum Product Assessment
WPM	Weighted Product Model
WSM	Weighted Sum Method
NIV	National Institute of Virology
WHO	World Health Organization
GBS	Guillain-Barre syndrome
PHEIC	Public Health Emergency of International Concern
ICMR	Indian Council of Medical Research
IFS	Intuitionistic Fuzzy Sets
TVFS	Interval-valued fuzzy sets
IVIFS	Interval-valued Intuitionistic Fuzzy Sets
HFS	Hesitant Fuzzy Sets
PFS	Pythagorean Fuzzy sets
T2PFS	Type-2 Pythagorean Fuzzy sets
TFS	Triangular Fuzzy Sets
TrFS	Trapezoidal Fuzzy Sets
CPFS	Complex Pythagorean Fuzzy sets
OFS	Orthopair Fuzzy sets
Q-OFS	Q-Rung Orthopair Fuzzy Sets
HeFS	Heptagonal Fuzzy Sets
T2HFS	Type-2 heptagonal fuzzy sets
T2HFWAO	Type-2 heptagonal fuzzy weighted arithmetic aggregation operator
T2HFPWAO	Type-2 heptagonal fuzzy power weighted arithmetic aggregation operator
T2HFWGO	Type-2 heptagonal fuzzy weighted geometric aggregation operator
T2HFPWGO	Type-2 heptagonal fuzzy power weighted geometric aggregation operator
T2HFOPWAO	Type-2 heptagonal fuzzy ordered power weighted arithmetic aggregation operator

## Preliminaries

Type-2 heptagonal fuzzy sets are initiated in this section. Primarily, a definition of type-1 fuzzy set and type-2 fuzzy set is given properly. To demonstrate the eventuality of the type-2 heptagonal fuzzy sets, an example is provided at the end of this section.

### Definition 1

A type-1 fuzzy set [13], $\tilde{X}$ in the universe of discourse *U* is characterized by a membership function $\mu _{a}(x)$ taking values on the interval [0, 1] and can be represented as a set of ordered pairs of an element to the set and are defined

$$\begin{aligned} X = \{a,\mu _{x}(a)|a \in X \} \end{aligned}$$

In this definition $\mu _{x}(a)$ represents the membership function of an element $a \in X$, to the set *X*

### Definition 2

A set $\overset{\approx }{H}$ is defined as a type-2 fuzzy set [53] in a universe of discourse *U* whose elements have secondary membership for the primary membership of every element in the set. A type-2 fuzzy number is defined as,

$$\begin{aligned} \overset{\approx }{H}=\left\{x, \mu _{\overset{\approx }{h_1}}(x), \mu _{\overset{\approx }{h_2}}(x)|x \in \overset{\approx }{U}\ \right\} \end{aligned}$$

Where, $\mu _{\overset{\approx }{h_1}}$ is primary membership value and $\mu _{\overset{\approx }{h_2}}$ is secondary membership value of the element *x*.

### Definition 3

A Heptagonal fuzzy set $\tilde{H}$ is defined [13] as a specific type of fuzzy set characterized by a membership function that maps elements to a heptagon-shaped fuzzy region. The membership function assigns each element in the universe of discourse a value between 0 and 1, representing its degree of membership in the set. The general form of the membership function for a heptagonal fuzzy set can be described as:

$$\begin{aligned} \mu _{\tilde{A}}(x) = \left\{ \begin{array}{ll} 0 & \text {if } x< a_1 \\ \frac{x - a_1}{a_2 - a_1} & \text {if } a_1 \le x< a_2 \\ \frac{x - a_2}{a_3 - a_2} & \text {if } a_2 \le x< a_3 \\ \frac{x - a_3}{a_4 - a_3} & \text {if } a_3 \le x< a_4 \\ 1 & \text {if } a_4 \le x \le a_5 \\ \frac{a_6 - x}{a_6 - a_5} & \text {if } a_5< x \le a_6 \\ \frac{a_7 - x}{a_7 - a_6} & \text {if } a_6 < x \le a_7 \\ 0 & \text {if } x> a_7 \end{array}\right. \end{aligned}$$

### Definition 4

A set $\overset{\approx }{H}$ is defined as a type-2 heptagonal fuzzy set in a universe of discourse *U* whose elements have type-2 membership value $\mu _{\overset{\approx }{H}}.$ A type-2 heptagonal fuzzy number is defined as,

$$\begin{aligned} \overset{\approx }{H}=\left\{x, \mu _{\overset{\approx }{h1}}(x), \mu _{\overset{\approx }{h2}}(x)|x \in \overset{\approx }{U}\ \right\} \end{aligned}$$

Here, $\mu _{\overset{\approx }{H}_{1}}(x) = \left( a_1^{p}, a_2^{p}, a_3^{p}, a_4^{p}, a_5^{p}, a_6^{p}, a_7^{p}\right)$ and $\mu _{\overset{\approx }{H}_{2}}(x) = \left( a_1^{s}, a_2^{s}, a_3^{s}, a_4^{s}, a_5^{s}, a_6^{s}, a_7^{s}\right)$ In this definition $\mu _{\overset{\approx }{h1}}(x)$ represents the primary membership of type-2 heptagonal fuzzy set and $\mu _{\overset{\approx }{h2}}(x)$ indicates the secondary membership of type-2 heptagonal fuzzy set.

**Fig. 4:**
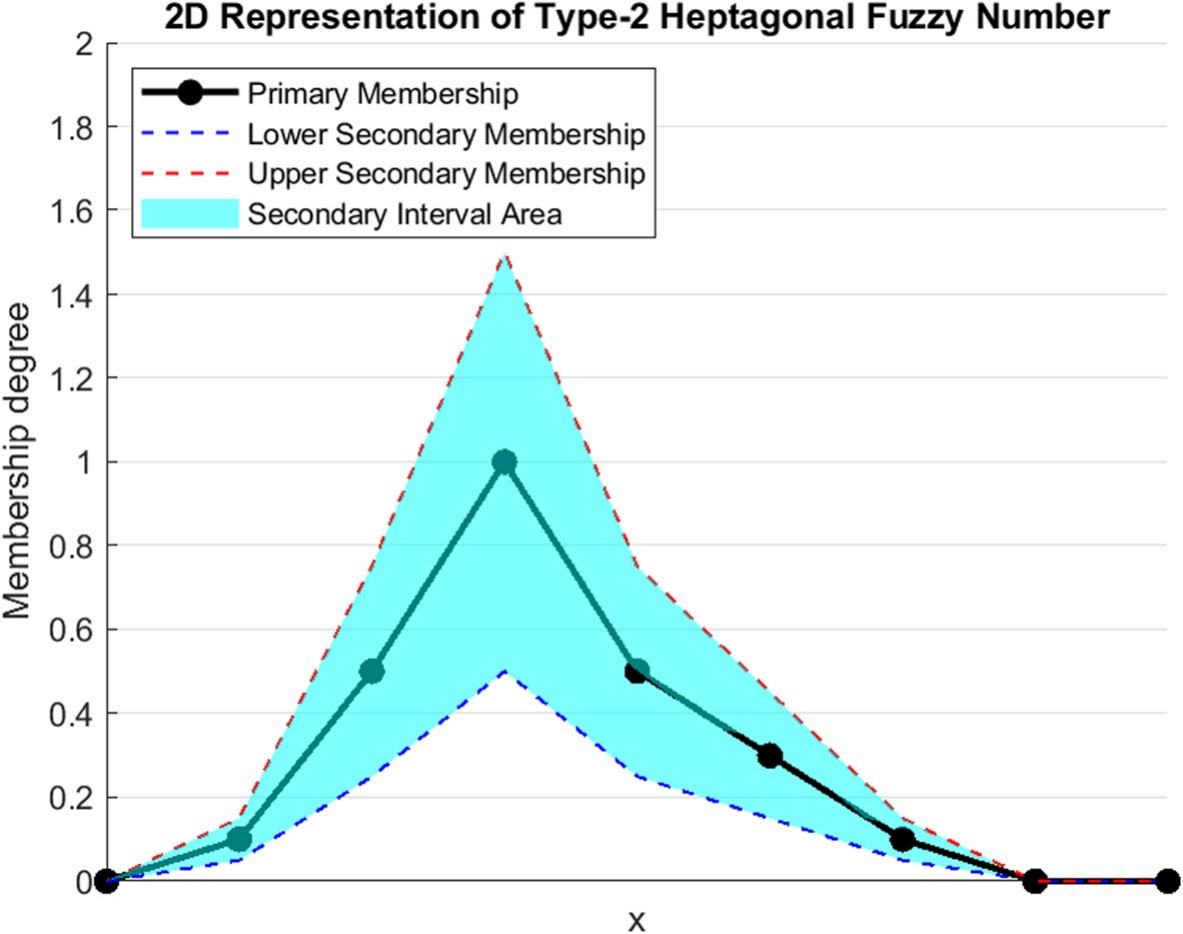
Graphical representation of type-2 heptagonal fuzzy number

**Fig. 5:**
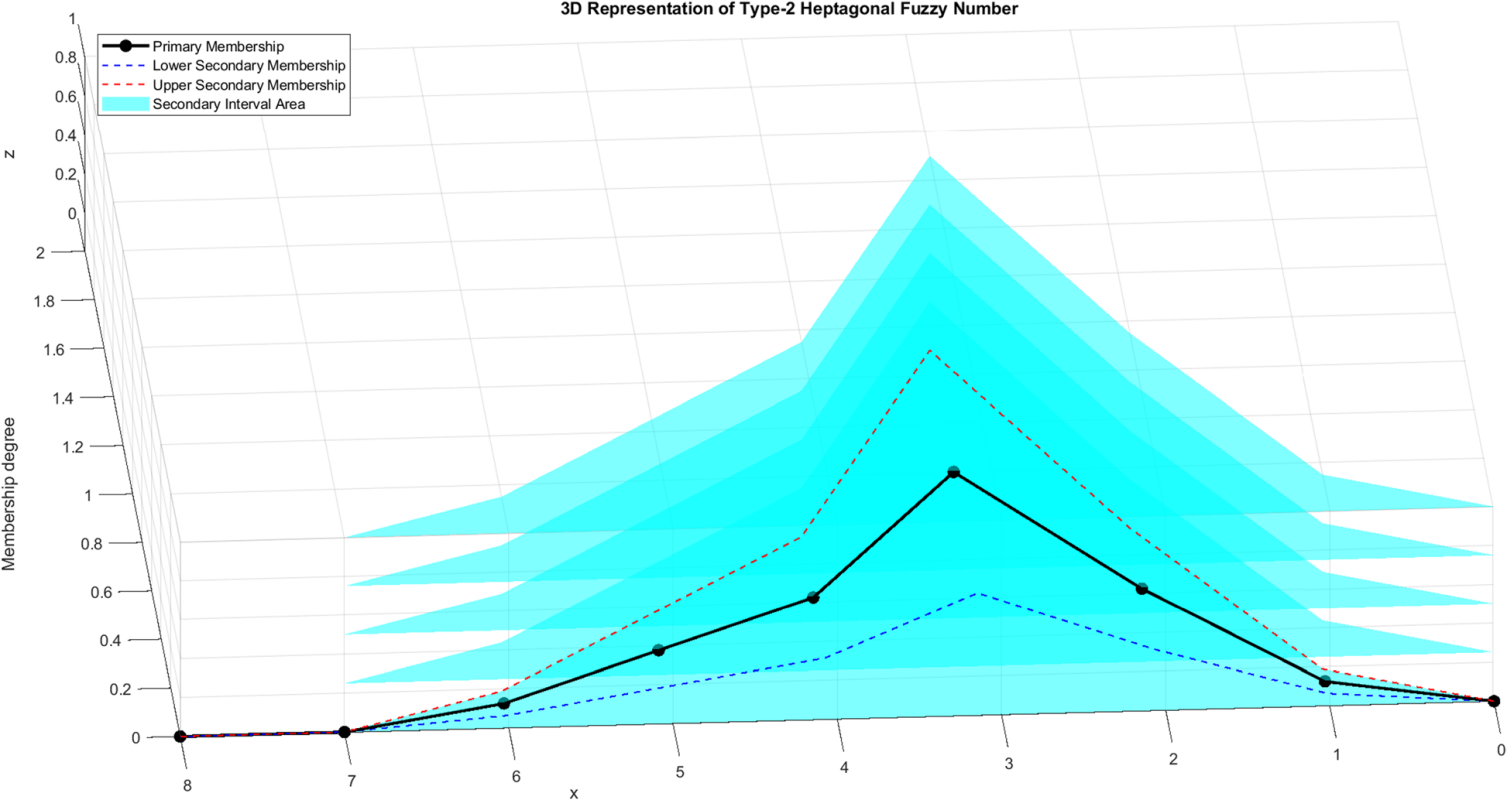
3D view of type-2 heptagonal fuzzy number

Figure 4 represents the view of type-2 heptagonal fuzzy number and Fig. 5 shows the 3-dimensional view of type-2 heptagonal fuzzy number.

### Score function

#### Definition 5

For a given $T2HFN \overset{\approx }{\alpha }=\left\{\left( a_1^{p}, a_2^{p}, a_3^{p}, a_4^{p}, a_5^{p}, a_6^{p}, a_7^{p}\right), \left(a_1^{s}, a_2^{s}, a_3^{s}, a_4^{s}, a_5^{s}, a_6^{s}, a_7^{s}\right) \right\},$ the score function is defined as,

1 $$\begin{aligned} \overset{\approx }{S}(H)=\sum \limits _{i=1}^{7}a_{i}^{p}a_{i}^{s} \end{aligned}$$

$$\begin{aligned} \overset{\approx }{S}(H)=\frac{1}{7}\left(a_1^{p}.a_1^{s}+a_2^{p}a_2^{s}+a_3^{p}a_3^{s}+a_4^{p}a_4^{s}+a_5^{p}a_5^{s}+a_6^{p}a_6^{s}+a_7^{p}a_7^{s}\right) \end{aligned}$$

This score function takes the arithmetic mean of all seven points, giving equal weight to each point.

#### Example

Consider a *T*2*HFNs* $\overset{\approx }{\alpha }=\{(0.12, 0.13, 0.21, 0.24, 0.31, 0.42, 0.47;1), (0.22, 0.43, 0.51, 0.64, 0.72, 0.79, 0.81;1)\}$, the score value of $\overset{\approx }{\alpha }$ is,

$$\begin{aligned} \overset{\approx }{S}(H) & =\frac{1}{7}[(0.12)(0.22)+(0.13)(0.43)+(0.21)(0.51)+(0.24)(0.64)+(0.31)(0.72)\\ & +(0.42)(0.79) +(0.47)(0.81)]\\ & = 0.183 \end{aligned}$$

##### Definition 6

For a given $T2HFN \overset{\approx }{\alpha }=\left\{ a_1, a_2, a_3, a_4, a_5, a_6, a_7\right\},$ the weighted score function is defined as,

2 $$\begin{aligned} \overset{\approx }{WS}(H)=\sum \limits _{i=1}^{7} w_i\left(a_{i}^{p}a_{i}^{s}\right) \end{aligned}$$

$$\begin{aligned} \overset{\approx }{WS}(H)=\frac{1}{7}\left(w_1\left(a_1^p.a_1^s\right)+w_2\left(a_2^p.a_2^s\right)+w_3\left(a_3^p.a_3^s\right)+w_4\left(a_4^p.a_4^s\right)+w_5\left(a_5^p.a_5^s\right)+w_6\left(a_6^p.a_6^s\right)+w_7\left(a_7^p.a_7^s\right)\right) \end{aligned}$$

#### Example

Let a *T*2*HFNs* $\overset{\approx }{\alpha }=\{(0.12, 0.13, 0.21, 0.24, 0.31, 0.42, 0.47), (0.22, 0.43, 0.51, 0.64, 0.72, 0.79, 0.81)\}$ and the weight is $\overset{\approx }{w}=\{ 0.05, 0.1, 0.12, 0.15, 0.18, 0.19, 0.21\}$, then $\overset{\approx }{WS}(H)$ is

$$\begin{aligned} \overset{\approx }{WS}(H) & = \frac{1}{7}(0.05(0.12*0.22)+0.1(0.13*0.43)+0.12(0.21*0.51)+0.15(0.24*0.64)+\\ & 0.18(0.31*0.72)+0.19(0.42*79)+0.21(0.47*0.81)\\ & = 0.226 \end{aligned}$$

### Accuracy function

#### Definition 7

For a provided *T*2*HFN* $\overset{\approx }{\alpha }=\left\{\left( a_1^{p}, a_2^{p}, a_3^{p}, a_4^{p}, a_5^{p}, a_6^{p}, a_7^{p}\right), \left(a_1^{s}, a_2^{s}, a_3^{s}, a_4^{s}, a_5^{s}, a_6^{s}, a_7^{s}\right) \right\}$, the accuracy function is defined as, The accuracy function lies in the interval $[-1, 1].$

$$\begin{aligned} Acc(\overset{\approx }{\alpha }) = \frac{\sum _{i=1}^{7} w_i \cdot \left( \frac{a_i^p + a_i^s}{2} \right) }{\sum _{i=1}^{7} \left( \frac{a_i^p+a_i^s}{2} \right) } \end{aligned}$$

where:

- $w_i$ are the weight values.
- $a_p$ and $a_s$ are the primary and secondary membership values at each point $a_i$.

### Example

Let a *T*2*HFNs* $\overset{\approx }{\alpha }=\{(0.12, 0.13, 0.21, 0.24, 0.31, 0.42, 0.47), (0.22, 0.43, 0.51, 0.64, 0.72, 0.79, 0.81)\}$ and the weight is $\overset{\approx }{w}=\{ 0.05, 0.1, 0.12, 0.15, 0.18, 0.19, 0.21\}$, then $Acc(\overset{\approx }{\alpha })$ is

$$\begin{aligned} Acc(\overset{\approx }{\alpha }) & = \frac{0.48775}{3.01000}\\ & = 0.162 \end{aligned}$$

### The t-norm and the t-conorm

#### Definition 8

Let us consider the membership functions of two *T*2*HFNs* provided by, $\overset{\approx }{\alpha }_{h}=\left\{\left(a_{h1}^p, a_{h2}^p, a_{h3}^p, a_{h4}^p, a_{h5}^p, a_{h6}^p, a_{h7}^p\right), \left(a_{h1}^s, a_{h2}^s, a_{h3}^s, a_{h4}^s, a_{h5}^s, a_{h6}^s, a_{h7}^s\right)\right\},$ and $\overset{\approx }{\beta }_{h}=\{\left(b_{h1}^p, b_{h2}^p, b_{h3}^p, b_{h4}^p, b_{h5}^p, b_{h6}^p, b_{h7}^p\right), \left(b_{h1}^s, b_{h2}^s, b_{h3}^s, b_{h4}^s, b_{h5}^s, b_{h6}^s, b_{h7}^s\right)\}$ then the t-norm and t-conorm defined as follows:

$$\begin{aligned} T(\overset{\approx }{\alpha }_{h}, \overset{\approx }{\beta }_{h}) & = \sum \limits _{i=1}^7 \left(a_{hi}^p.b_{hi}^p.a_{hi}^s.b_{hi}^s\right)\\ S(\overset{\approx }{\alpha }_{h}, \overset{\approx }{\beta }_{h}) & = a_{hi}^p.a_{hi}^s+b_{hi}^p.b_{hi}^s-a_{hi}^p.a_{hi}^s* b_{hi}^p.b_{hi}^s \end{aligned}$$

The t-norm satisfies the following axioms.

1. Commutativity $$T(\overset{\approx }{\alpha }_{h}, \overset{\approx }{\beta }_{h}) = T(\overset{\approx }{\beta }_{h}, \overset{\approx }{\alpha }_{h})$$
2. Associativity $$T(\overset{\approx }{\alpha }_h, T(\overset{\approx }{\beta }_{h}, \overset{\approx }{\gamma }_{h})) = T(T(\overset{\approx }{\alpha }_{h},\overset{\approx }{\beta }_{h}), (\overset{\approx }{\gamma }_{h}))$$
3. Boundary conditions $$T(\overset{\approx }{\alpha }_{h}, 0) = 0$$
4. Monotonocity $$|T(\overset{\approx }{\alpha }_{h}, \overset{\approx }{\beta }_{h})| \le |T(\overset{\approx }{\alpha }_{h}, \overset{\approx }{\gamma }_{h})|$$ when $$|\overset{\approx }{\beta }_{h}| \le |\overset{\approx }{\gamma }_{h}|$$

The t- conorm satisfies the following axioms

1. Commutativity $$S(\overset{\approx }{\alpha }_{h}, \overset{\approx }{\beta }_{h}) = S(\overset{\approx }{\beta }_{h}, \overset{\approx }{\alpha }_{h})$$
2. Associativity $$S(\overset{\approx }{\alpha }_{h}, S(\overset{\approx }{\beta }_{h}, \overset{\approx }{\gamma }_{h})) = S(S(\overset{\approx }{\alpha }_{h},\overset{\approx }{\beta }_{h}), (\overset{\approx }{\gamma }_{h}))$$
3. Boundary conditions $$S(\overset{\approx }{\alpha }_{h}, 0) = \overset{\approx }{\alpha }_{h}$$
4. Monotono city $|S(\overset{\approx }{\alpha }_{h}, \overset{\approx }{\beta }_{h})| \le |S(\overset{\approx }{\alpha }_{h}, \overset{\approx }{\gamma }_{h})|$ when $|\overset{\approx }{\beta }_{h}| \le |\overset{\approx }{\gamma }_{h}|$

### Union

#### Definition 9

Take two *T*2*HFS*, $\overset{\approx }{H_{\alpha }}$ and $\overset{\approx }{H_{\beta }}$. type-2 heptagonal fuzzy union is represented as, $\overset{\approx }{H_{\alpha }} \cup \overset{\approx }{H_{\beta }}$ which is described by the function $\overset{\approx }{f}:\overset{\approx }{H}_{\alpha }({x}) \times \overset{\approx }{H}_{\beta }(x) \mapsto \overset{\approx }{H}_{\gamma }(x)$.

Following axioms are the fundamental requirements of the type-2 heptagonal fuzzy union function:

1. Boundary conditions: $$\overset{\approx }{f}(\overset{\approx }{H}_{\alpha },0)=\overset{\approx }{H}_{\alpha }$$
2. Monotonicity: $$|\overset{\approx }{H}_{\alpha }|\le |\overset{\approx }{H}_{\gamma }| \Rightarrow |\overset{\approx }{f}(\overset{\approx }{H}_{\beta },\overset{\approx }{H}_{\alpha })| \le |\overset{\approx }{f}(\overset{\approx }{H}_{\beta },\overset{\approx }{H}_{\gamma })|$$
3. Commutativity: $$\overset{\approx }{f}(\overset{\approx }{H}_{\alpha }, \overset{\approx }{H}_{\beta }) = \overset{\approx }{f}(\overset{\approx }{H}_{\beta }, \overset{\approx }{H}_{\alpha })$$
4. Associativity: $$\overset{\approx }{f}(\overset{\approx }{H}_{\alpha }, \overset{\approx }{f}(\overset{\approx }{H}_{\beta }, \overset{\approx }{H}_{\gamma }))= \overset{\approx }{f}(\overset{\approx }{f}\overset{\approx }{H}_{\alpha }, \overset{\approx }{H}_{\beta }), \overset{\approx }{H}_{\gamma })$$

The type-2 heptagonal fuzzy union is represented as

$$\begin{aligned} \overset{\approx }{H}_{\alpha } \cup \overset{\approx }{H}_{\beta }=\{\textrm{max}(a_{hi}^p, b_{hi}^p), \textrm{max}(a_{hi}^s, b_{hi}^s) \} \end{aligned}$$

#### Example

Let us consider $\overset{\approx }{H}_{\alpha } = \{(0.12, 0.15, 0.23, 0.33, 0.45, 0.6, 0.69), (0.24, 0.27, 0.32, 0.34, 0.46, 0.58, 0.71) \}$ and

$$\overset{\approx }{H}_{\beta } = \{(0.22, 0.27, 0.33, 0.37, 0.42, 0.48, 0.56, 0.68), (0.29, 0.39, 0.43, 0.47, 0.51, 0.63, 0.76) \}$$ using the

maximum function is given as,

$$\begin{aligned} \overset{\approx }{H}_{\alpha } \cup \overset{\approx }{H}_{\beta } & = \{(0.12, 0.15, 0.23, 0.33, 0.45, 0.6, 0.69), (0.24, 0.27, 0.32, 0.34, 0.46, 0.58, 0.71) \} \cup \\ & \{(0.22, 0.27, 0.33, 0.37, 0.42, 0.48, 0.56, 0.68), (0.29, 0.39, 0.43, 0.47, 0.51, 0.63, 0.76) \}\\ & = \{(0.22, 0.27, 0.33, 0.37, 0.45, 0.6, 0.69), (0.29, 0.39, 0.43, 0.47, 0.51, 0.63, 0.76) \} \end{aligned}$$

### Intersection

#### Definition 10

Take two *T*2*HFS*, $\overset{\approx }{H_{\alpha }}$ and $\overset{\approx }{H_{\beta }}$. The type-2 heptagonal fuzzy intersection is represented as, $\overset{\approx }{H_{\alpha }} \cap \overset{\approx }{H_{\beta }}$ which is described by the function $\overset{\approx }{f}:\overset{\approx }{H}_{\alpha }({x}) \times \overset{\approx }{H}_{\beta }(x) \mapsto \overset{\approx }{H}_{\gamma }(x)$.

Following axioms are the fundamental requirements of the type-2 heptagonal fuzzy intersection function:

1. Boundary conditions: $$\overset{\approx }{f}(\overset{\approx }{H}_{\alpha },0)=\overset{\approx }{H}_{\alpha }$$
2. Monotono city: $$|\overset{\approx }{H}_{\alpha }|\le |\overset{\approx }{H}_{\gamma }| \Rightarrow |\overset{\approx }{u}(\overset{\approx }{H}_{\beta },\overset{\approx }{H}_{\alpha })| \le |\overset{\approx }{u}(\overset{\approx }{H}_{\beta },\overset{\approx }{H}_{\gamma })|$$
3. Commutativity: $$\overset{\approx }{f}(\overset{\approx }{H}_{\alpha }, \overset{\approx }{H}_{\beta }) = \overset{\approx }{f}(\overset{\approx }{H}_{\beta }, \overset{\approx }{H}_{\alpha })$$
4. Associativity: $$\overset{\approx }{f}(\overset{\approx }{H}_{\alpha }, \overset{\approx }{f}(\overset{\approx }{H}_{\beta }, \overset{\approx }{H}_{\gamma }))= \overset{\approx }{f}(\overset{\approx }{f}\overset{\approx }{H}_{\alpha }, (\overset{\approx }{H}_{\beta }, \overset{\approx }{H}_{\gamma }))$$

The type-2 heptagonal fuzzy intersection is represented as

$$\begin{aligned} \overset{\approx }{H}_{\alpha } \cap \overset{\approx }{H}_{\beta }=\left\{\min \left(a_{hi}^p, b_{hi}^p\right), \min \left(a_{hi}^s, b_{hi}^s\right)\right \} \end{aligned}$$

#### Example

Let $\overset{\approx }{H}_{\alpha } = \{(0.12, 0.15, 0.23, 0.33, 0.45, 0.6, 0.69), (0.24, 0.27, 0.32, 0.34, 0.46, 0.58, 0.71) \}$ and $\overset{\approx }{H}_{\beta } = \{(0.22, 0.27, 0.33, 0.37, 0.42, 0.48, 0.56, 0.68), (0.29, 0.39, 0.43, 0.47, 0.51, 0.63, 0.76) \}$ using the minimum function is given as,

$$\begin{aligned} \overset{\approx }{H}_{\alpha } \cap \overset{\approx }{H}_{\beta } & = \{(0.12, 0.15, 0.23, 0.33, 0.45, 0.6, 0.69), (0.24, 0.27, 0.32, 0.34, 0.46, 0.58, 0.71) \} \cap \\ & \{(0.22, 0.27, 0.33, 0.37, 0.42, 0.48, 0.56), (0.29, 0.39, 0.43, 0.47, 0.51, 0.63, 0.76) \}\\ & = \{(0.12, 0.15, 0.23, 0.33, 0.42, 0.48, 0.56), (0.24, 0.27, 0.32, 0.34, 0.46, 0.58, 0.71) \} \end{aligned}$$

### Complement

#### Definition 11

Consider a *T*2*HFS* $\overset{\approx }{H}_{\alpha }$. The type-2 heptagonal fuzzy complement is described by a function $\overset{\approx }{f}:\overset{\approx }{H}_{\alpha }({x}) \mapsto \overset{\approx }{H}_{\alpha }^{c}(x)$.

1. Boundary conditions: $|\overset{\approx }{\alpha }(x)|=0 \Longrightarrow |\overset{\approx }{c}(\overset{\approx }{\alpha })(\overset{\approx }{x})|=1$ and $|\hat{\alpha }(x)|=1 \Longrightarrow |\overset{\approx }{c}(\overset{\approx }{\alpha })(\overset{\approx }{x})|=0$
2. Monotonicity: If $|\overset{\approx }{\alpha }(\overset{\approx }{x})| \le |\overset{\approx }{\alpha }(\overset{\approx }{x})|$ then $|c(\overset{\approx }{\alpha }(\overset{\approx }{x}))| \ge |c(\overset{\approx }{\alpha }(\hat{x}))|$

The type-2 heptagonal fuzzy complement is represented as,

$$\begin{aligned} \overset{\approx }{H}_{\alpha } & = \left\{\left(a_{h1}^p, a_{h2}^p, a_{h3}^p, a_{h4}^p, a_{h5}^p, a_{h6}^p, a_{h7}^p\right), \left(a_{h1}^s, a_{h2}^s, a_{h3}^s, a_{h4}^s, a_{h5}^s, a_{h6}^s, a_{h7}^s\right)\right\}\\ \overset{\approx }{H}_{\alpha }^c & = \left\{\left(1-a_{h1}^p, 1-a_{h2}^p, 1-a_{h3}^p, 1-a_{h4}^p, 1-a_{h5}^p, 1-a_{h6}^p, 1-a_{h7}^p\right),\right.\\ & \left.\left(1-a_{h1}^s, 1-a_{h2}^s, 1-a_{h3}^s, 1-a_{h4}^s, 1-a_{h5}^s, 1-a_{h6}^s, 1-a_{h7}^s\right)\right\} \end{aligned}$$

#### Example

Let $\overset{\approx }{H}_{\alpha } = \{(0.12, 0.15, 0.23, 0.33, 0.45, 0.6, 0.69), (0.24, 0.27, 0.32, 0.34, 0.46, 0.58, 0.71) \}$ and $\overset{\approx }{H}_{\alpha }^c$ is,

$$\begin{aligned} \overset{\approx }{H}_{\alpha } & = \{(0.12, 0.15, 0.23, 0.33, 0.45, 0.6, 0.69), (0.24, 0.27, 0.32, 0.34, 0.46, 0.58, 0.71) \}\\ \overset{\approx }{H}_{\alpha }^{c} & = \{(0.88, 0.85, 0.77, 0.67, 0.55, 0.4, 0.31), (0.76, 0.73, 0.68, 0.66, 0.54, 0.42, 0.29) \} \end{aligned}$$

## Type-2 heptagonal fuzzy arithmetic aggregation operator

In this section, type-2 heptagonal fuzzy weighted arithmetic aggregation operator, and type-2 heptagonal fuzzy powered arithmetic aggregation operator are explained with examples.

### Type-2 heptagonal fuzzy weighted arithmetic aggregation operator

Type-2 heptagonal fuzzy arithmetic aggregation operator is defined here with various properties.

#### Definition 12

On a universe of discourse $\overset{\approx }{X}$, let us consider a set of *m* *T*2*HFNs* $\overset{\approx }{\alpha }_{h1}, \overset{\approx }{\alpha }_{h2}, \overset{\approx }{\alpha }_{h3}, \ldots \overset{\approx }{\alpha }_{hm}$. The aggregation of the vectors $\overset{\approx }{\alpha }_{h1}, \overset{\approx }{\alpha }_{h2}, \overset{\approx }{\alpha }_{h3}, \ldots \overset{\approx }{\alpha }_{hm}$ is provided by the function *T*2*HFWA*

$$\begin{aligned} T2HFWA : \overset{\approx }{X}^{n} \mapsto \overset{\approx }{X} \end{aligned}$$

The operator is defined as,

$$\begin{aligned} T2HFWA\left(\overset{\approx }{\alpha }_{h1}, \overset{\approx }{\alpha }_{h2}, \overset{\approx }{\alpha }_{h3}, \ldots \overset{\approx }{\alpha }_{hm}\right)= \sum \limits _{j=1}^{m} \vec {\delta }_{j} \overset{\approx }{\alpha }_{hj} \end{aligned}$$

where, $\vec {\delta }=\left(\vec {\delta }_{1}, \vec {\delta }_{2}, \vec {\delta }_{3}, \ldots , \vec {\delta }_{m}\right)$ is the weight vector in which $\vec {\delta }_{j} \in [0,1]$ and $\sum _{j=1}^{m}|\vec {\delta }_{j}|=1$. Weights are numeral quantities and are utilized in this definition in general.

$$\begin{aligned} T2HFWA\left(\overset{\approx }{\alpha }_{h1}, \overset{\approx }{\alpha }_{h2}, \overset{\approx }{\alpha }_{h3}, \ldots \overset{\approx }{\alpha }_{hm}\right)=\sum \limits _{j=1}^{m} \vec {\delta }_{j}\left[\overset{\approx }{\alpha _{hj}}^p,\overset{\approx }{\alpha _{hj}}^s\right] \end{aligned}$$

#### Theorem 1

Consider *m* *T*2*HFNs* denoted by $\overset{\approx }{\alpha }_{h1}, \overset{\approx }{\alpha }_{h2}, \overset{\approx }{\alpha }_{h3}, \ldots \overset{\approx }{\alpha }_{hm}$. The value obtained after aggregating these *m* *T*2*HFNs* by the *T*2*HFWA* operator is again a *T*2*HFN*.

#### Proof

Seeing that $|\overset{\approx }{\alpha }_{hj}|\le 1 \forall j=1,2,\ldots ,m$ and $\sum _{j=1}^{m}\vec {\delta }_{j}=1.$ It obtained as follows,

$$\begin{aligned} |\vec {\delta }_{1}\overset{\approx }{\alpha }_{h1}+\vec {\delta }_{2}\overset{\approx }{\alpha }_{h2}+\vec {\delta }_{3}\overset{\approx }{\alpha }_{h3}+\ldots +\vec {\delta }_{m}\overset{\approx }{\alpha }_{hm}| & \le \vec {\delta }_{1}|\overset{\approx }{\alpha }_{h1}|+\vec {\delta }_{2}|\overset{\approx }{\alpha }_{h2}|+\vec {\delta }_{3}|\overset{\approx }{\alpha }_{h3}| + \dots + \vec {\delta }_{m}|\overset{\approx }{\alpha }_{cm}|\\ & \le \vec {\delta }_{1}(1)+\vec {\delta }_{2}(1)+\vec {\delta }_{3}(1)+\ldots +\vec {\delta }_{m}(1)\\ & \le \vec {\delta }_{1}+\vec {\delta }_{2}+\vec {\delta }_{3}+\ldots +\vec {\delta }_{m}\\ & =1 \end{aligned}$$

Hence it is clear that $|T2HFWA\left(\overset{\approx }{\alpha }_{h1}, \overset{\approx }{\alpha }_{h2}, \overset{\approx }{\alpha }_{h3}, \ldots \overset{\approx }{\alpha }_{hm}\right)|\le 1$ is also *T*2*HFN* $\square$

#### Theorem 2

Take *m* *T*2*HFNs* which have weights to be equal valued that satisfy $\vec {\delta }_{j}=1$. Then following holds,

1. (Idempotency) When $\overset{\approx }{\alpha }_{h1}=\overset{\approx }{\alpha }_{h2}=\overset{\approx }{\alpha }_{h3}= \ldots = \overset{\approx }{\alpha }_{hm}=\overset{\approx }{\alpha },$ then the operator becomes $T2HFWA\left(\overset{\approx }{\alpha }_{h1}, \overset{\approx }{\alpha }_{h2}, \overset{\approx }{\alpha }_{h3}, \ldots \overset{\approx }{\alpha }_{hm}\right)=\overset{\approx }{\alpha }$
2. (Boundedness) Consider $\textrm{max}_{j}|\overset{\approx }{\alpha }_{hj}|,$ then $|T2HFWA\left(\overset{\approx }{\alpha }_{h1}, \overset{\approx }{\alpha }_{h2}, \overset{\approx }{\alpha }_{h3}, \ldots \overset{\approx }{\alpha }_{hm}\right)| \le a$
3. (Monotonocity) Consider two sets of $T2HFWA \left\{ \overset{\approx }{\alpha }_{h1}, \overset{\approx }{\alpha }_{h2}, \overset{\approx }{\alpha }_{h3}, \ldots \overset{\approx }{\alpha }_{hm} \right\}$ and $\left\{ \overset{\approx }{\beta }_{h1}, \overset{\approx }{\beta }_{h2}, \overset{\approx }{\beta }_{h3}, \ldots \overset{\approx }{\beta }_{hm} \right\}$ such that $|\overset{\approx }{\alpha }_{h1}|\le |\overset{\approx }{\beta }_{h1}|, |\overset{\approx }{\alpha }_{h2}|\le |\overset{\approx }{\beta }_{h2}|, \ldots , |\overset{\approx }{\alpha }_{hm}|\le |\overset{\approx }{\beta }_{hm}|$, then the aggregation operator can be written as, $|T2HFWA\left(\overset{\approx }{\alpha }_{h1}, \overset{\approx }{\alpha }_{h2}, \overset{\approx }{\alpha }_{h3}, \ldots , \overset{\approx }{\alpha }_{hm}\right)| = |T2HFWA\left(\overset{\approx }{\beta }_{h1}, \overset{\approx }{\beta }_{h2}, \overset{\approx }{\beta }_{h3}, \ldots , \overset{\approx }{\beta }_{hm}\right)|$

#### Proof

By the given conditions,

1. As $\overset{\approx }{\alpha }_{h1}=\overset{\approx }{\alpha }_{h2}=\overset{\approx }{\alpha }_{h3}=\ldots =\overset{\approx }{\alpha }_{hm}=\overset{\approx }{\alpha }$ and $\sum _{j=1}^{m}\vec {\delta }_{j}=1$, hence
  $$\begin{aligned} & = \overset{\approx }{\alpha }_{h1}\vec {\delta }_{1}+\overset{\approx }{\alpha }_{h2}\vec {\delta }_{2}+\ldots +\overset{\approx }{\alpha }_{hm}\vec {\delta }_{m}\\ & = \left(\vec {\delta }_{1}+\vec {\delta }_{2}+\ldots +\vec {\delta }_{m}\right)\overset{\approx }{\alpha }_{hm}\\ & = \overset{\approx }{\alpha } \end{aligned}$$
2. As $$a=\textrm{max}_{j}|\overset{\approx }{\alpha }_{hj}|$$ and $$\vec {\delta }_{j}\in [0,1] \forall j=1,2,\ldots ,m$$
3. Here, $$|\overset{\approx }{\alpha }_{h1}| \le |\overset{\approx }{\beta }_{h1}|, |\overset{\approx }{\alpha }_{h1}| \le |\overset{\approx }{\beta }_{h1}|, |\overset{\approx }{\alpha }_{h2}| \le |\overset{\approx }{\beta }_{h2}|, \ldots , |\overset{\approx }{\alpha }_{hm}| \le |\overset{\approx }{\beta }_{hm}|.$$ Hence,
  $$\begin{aligned} |\vec {\delta }_{1}\overset{\approx }{\alpha }_{h1}+\vec {\delta }_{2}\overset{\approx }{\alpha }_{h2}+\vec {\delta }_{3}\overset{\approx }{\alpha }_{h3}+\ldots +\vec {\delta }_{m}\overset{\approx }{\alpha }_{hm}| & \le \vec {\delta }_{1}|\overset{\approx }{\alpha }_{h1}|+\vec {\delta }_{2}|\overset{\approx }{\alpha }_{h2}|+\vec {\delta }_{3}|\overset{\approx }{\alpha }_{h3}|+\ldots +\vec {\delta }_{m}|\overset{\approx }{\alpha }_{hm}|\\ & \le \vec {\delta }_{1}|\overset{\approx }{\beta }_{h1}|+\vec {\delta }_{2}|\overset{\approx }{\beta }_{h2}|+\vec {\delta }_{3}|\overset{\approx }{\beta }_{h3}|+\ldots +\vec {\delta }_{m}|\overset{\approx }{\beta }_{hm}|\\ T2HFWA\left(\overset{\approx }{\alpha }_{h1}, \overset{\approx }{\alpha }_{h2}, \overset{\approx }{\alpha }_{h3}, \ldots , \overset{\approx }{\alpha }_{hm}\right) & \le T2HFWA\left(\overset{\approx }{\beta }_{h1}, \overset{\approx }{\beta }_{h2}, \overset{\approx }{\beta }_{h3}, \ldots , \overset{\approx }{\beta }_{hm}\right) \end{aligned}$$

$$\square$$

#### Example

Consider $\overset{\approx }{\alpha }_{h1}= \{(0.01, 0.12, 0.15, 0.18, 0.21, 0.25, 0.32), (0.22, 0.25, 0.29, 0.32, 0.37, 0.41, 0.43, 0.5)\}, \overset{\approx }{\alpha }_{h2}=\{(0.05, 0.14, 0.16, 0.22, 0.23, 0.27, 0.36), (0.20, 0.22, 0.25, 0.28, 0.30, 0.32, 0.35, 0.42)\}, \overset{\approx }{\alpha }_{h3}= \{(0.1, 0.15, 0.19, 0.24, 0.26, 0.31, 0.34), (0.15, 0.19, 0.29, 0.32, 0.37, 0.43, 0.45, 0.5)\}$ and $\overset{\approx }{\alpha }_{h4}= \{(0.12, 0.15, 0.19, 0.21, 0.27, 0.29, 0.32), (0.16, 0.25, 0.27, 0.32, 0.39, 0.41, 0.43, 0.5)\}$ having weights

$\vec {\delta }_{1}=0.1, \vec {\delta }_{2}=0.3, \vec {\delta }_{3}=0.25, \vec {\delta }_{4}=0.35$, then the sum obtained by *T*2*HFWA* operator is,

$$\begin{aligned} T2HFWA\left(\overset{\approx }{\alpha }_{h1}, \overset{\approx }{\alpha }_{h2}, \overset{\approx }{\alpha }_{h3}, \overset{\approx }{\alpha }_{h4}\right) & = \sum \limits _{j=1}^{4} \delta _{j}\left(\overset{\approx }{\alpha }_{j}\right)\\ & = 0.1\{(0.01, 0.12, 0.15, 0.18, 0.21, 0.25, 0.32),\\ & (0.22, 0.25, 0.29, 0.32, 0.37, 0.41, 0.43, 0.5)\}\\ & +0.25\{(0.05, 0.14, 0.16, 0.22, 0.23, 0.27, 0.36),\\ & (0.20, 0.22, 0.25, 0.28, 0.30, 0.32, 0.35,)\}\\ & +0.3\{(0.1, 0.15, 0.19, 0.24, 0.26, 0.31, 0.34), \\ & (0.15, 0.19, 0.29, 0.32, 0.37, 0.43, 0.45, 0.5)\}\\ & +0.35\{(0.12, 0.15, 0.19, 0.21, 0.27, 0.29, 0.32),\\ & (0.16, 0.25, 0.27, 0.32, 0.39, 0.41, 0.43, 0.5)\}\\ & = \{(0.08, 0.14, 0.18, 0.22, 0.25, 0.29, 0.34), \\ & (0.18, 0.23, 0.27, 0.31, 0.36, 0.39, 0.41, 0.48)\} \end{aligned}$$

### Type-2 heptagonal fuzzy ordered weighted arithmetic aggregation operator

#### Definition 13

On a universe of discourse $\overset{\approx }{X}$, let us consider a set of *m* *T*2H*FNs*, $\overset{\approx }{\alpha }_{h1}, \overset{\approx }{\alpha }_{h2}, \overset{\approx }{\alpha }_{h3}, \ldots \overset{\approx }{\alpha }_{hm}$, the aggregation of the vectors $\overset{\approx }{\alpha }_{h1}, \overset{\approx }{\alpha }_{h2}, \overset{\approx }{\alpha }_{h3}, \ldots \overset{\approx }{\alpha }_{hm}$ is provided by the function *T*2*HFOWA*, 

$$\begin{aligned} T2HFOWA : \overset{\approx }{X}^{n} \mapsto \overset{\approx }{X} \end{aligned}$$

The operator is defined as,

$$\begin{aligned} T2HFOWA\left(\overset{\approx }{\alpha }_{h1}, \overset{\approx }{\alpha }_{h2}, \overset{\approx }{\alpha }_{h3}, \ldots \overset{\approx }{\alpha }_{hm}\right)=\sum \limits _{j=1}^{m}\left(\vec {\delta }\overset{\approx }{\alpha }_{co(j)}\right) \end{aligned}$$

Where $\vec {\delta }=\left(\vec {\delta }_{1}, \vec {\delta }_{2}, \vec {\delta }_{3}, \ldots , \vec {\delta }_{m}\right)$ is the weight vector in which $\vec {\delta }_{j} \in [0,1]$ and $\sum _{j=1}^{m}|\vec {\delta }_{j}|=1$ and $(o(1), o(2), o(3), \ldots , o(m))$ is the permutation of elements such that $|\overset{\approx }{\alpha }_{ohj}| \le |\overset{\approx }{\alpha }_{oh(j-1)}|$. Weights which are normal quantities utilised in this definition, so the definition is more general. The aggregation operator can also be written as,

$$\begin{aligned} T2HFOWA\left(\overset{\approx }{\alpha }_{h1}, \overset{\approx }{\alpha }_{h2}, \overset{\approx }{\alpha }_{h3}, \ldots \overset{\approx }{\alpha }_{hm}\right)=\sum \limits _{j=1}^{m}\vec {\delta }_{j}\left[\overset{\approx }{\alpha }_{o(hj)}^p, \overset{\approx }{\alpha }_{o(hj)}^s\right] \end{aligned}$$

If the weight consider as $\vec {\delta }=\frac{1}{n}$, then *T*2*HFOWA* is given by,

3 $$\begin{aligned} T2HFOWA\left(\overset{\approx }{\alpha }_{h1}, \overset{\approx }{\alpha }_{h2}, \overset{\approx }{\alpha }_{h3}, \ldots \overset{\approx }{\alpha }_{hm}\right)=\frac{1}{n}\sum \limits _{j=1}^{m}\overset{\approx }{\alpha }_{{o(hj)}} \end{aligned}$$

#### Theorem 3

Consider the *m* *T*2*HFNs* denoted by $\left(\overset{\approx }{\alpha }_{h1}, \overset{\approx }{\alpha }_{h2}, \overset{\approx }{\alpha }_{h3}, \ldots \overset{\approx }{\alpha }_{hm}\right),$ the value obtained after aggregating these *m* *T*2*HFNs* by the *T*2*HFOW* operator is again a *T*2*HFN*

#### Proof

The proof is consequent to Theorem 1, hence omitted. $\square$

#### Theorem 4

Consider $\left({\overset{\approx }{\alpha }}_{h1}, {\overset{\approx }{\alpha }}_{h2}, {\overset{\approx }{\alpha }}_{h3}, \ldots {\overset{\approx }{\alpha }}_{hm}\right)$ to be *m* $T2HFN's$ and the weight values to be real values $\vec {\delta }=\vec {\delta }_{1}, \vec {\delta }_{2}, \ldots , \vec {\delta }_{m}$ and $|\vec {\delta }_{j}| \le 1$, then the following holds

1. (Idempotency) When $\overset{\approx }{\alpha }_{h1}=\overset{\approx }{\alpha }_{h2}=\overset{\approx }{\alpha }_{h3}= \ldots = \overset{\approx }{\alpha }_{hm}=\overset{\approx }{\alpha },$ then the operator becomes $T2HFOWA\left(\overset{\approx }{\alpha }_{h1}, \overset{\approx }{\alpha }_{h2}, \overset{\approx }{\alpha }_{h3}, \ldots \overset{\approx }{\alpha }_{hm}\right)=\overset{\approx }{\alpha }$
2. (Boundedness) Consider $\textrm{max}_{j}|\overset{\approx }{\alpha }_{hj}|,$ then $|T2HFWA\left(\overset{\approx }{\alpha }_{h1}, \overset{\approx }{\alpha }_{h2}, \overset{\approx }{\alpha }_{h3}, \ldots \overset{\approx }{\alpha }_{hm}\right)| \le a$
3. (Monotonocity) Consider two sets of $T2HFOWA \left\{ \overset{\approx }{\alpha }_{h1}, \overset{\approx }{\alpha }_{h2}, \overset{\approx }{\alpha }_{h3}, \ldots \overset{\approx }{\alpha }_{hm} \right\}$ and $\left\{ \overset{\approx }{\beta }_{h1}, \overset{\approx }{\beta }_{h2}, \overset{\approx }{\beta }_{h3}, \ldots \overset{\approx }{\beta }_{hm} \right\}$ such that $|\overset{\approx }{\alpha }_{h1}|\le |\overset{\approx }{\beta }_{h1}|, |\overset{\approx }{\alpha }_{h2}|\le |\overset{\approx }{\beta }_{h2}|, \ldots , |\overset{\approx }{\alpha }_{hm}|\le |\overset{\approx }{\beta }_{hm}|$, then the aggregation operator can be written as, $\left|T2HOWA\left(\overset{\approx }{\alpha }_{h1}, \overset{\approx }{\alpha }_{h2}, \overset{\approx }{\alpha }_{h3}, \ldots , \overset{\approx }{\alpha }_{hm}\right)| = |T2HFOWA\left(\overset{\approx }{\beta }_{h1}, \overset{\approx }{\beta }_{h2}, \overset{\approx }{\beta }_{h3}, \ldots , \overset{\approx }{\beta }_{hm}\right)\right|$

#### Proof

The proof is consequent to Theorem 2, hence omitted. $\square$

#### Example

Consider ${\overset{\approx }{\alpha }}_{h1}= \{(0.01, 0.12, 0.15, 0.18, 0.21, 0.25, 0.32), (0.22, 0.25, 0.29, 0.32, 0.37, 0.41, 0.43, 0.5)\}, \overset{\approx }{\alpha }_{h2}=\{(0.05, 0.14, 0.16, 0.22, 0.23, 0.27, 0.36), (0.20, 0.22, 0.25, 0.28, 0.30, 0.32, 0.35, 0.42)\}, \overset{\approx }{\alpha }_{h3}= \{(0.1, 0.15, 0.19, 0.24, 0.26, 0.31, 0.34), (0.15, 0.19, 0.29, 0.32, 0.37, 0.43, 0.45, 0.5)\}$ and $\overset{\approx }{\alpha }_{h4}= \{(0.12, 0.15, 0.19, 0.21, 0.27, 0.29, 0.32), (0.16, 0.25, 0.27, 0.32, 0.39, 0.41, 0.43, 0.5)\}$, then the sum obtained by *T*2*HFOWA* operator is, by Eq. 3, here $|\overset{\approx }{\alpha }_{h3}| \ge |\overset{\approx }{\alpha }_{h4}| \ge |\overset{\approx }{\alpha }_{h1}| \ge |\overset{\approx }{\alpha }_{h2}|$

$$\begin{aligned} T2HFOWA\left(\overset{\approx }{\alpha }_{h1}, \overset{\approx }{\alpha }_{h2}, \overset{\approx }{\alpha }_{h3}, \overset{\approx }{\alpha }_{h4}\right) & = \sum \limits _{j=1}^{4} \delta _{j}\left(\overset{\approx }{\alpha }_{o(j)}\right)\\ & = \{(0.078, 0.14, 0.18, 0.21, 0.25, 0.29,0.33), \\ & (0.18, 0.23, 0.28, 0.32, 0.37, 0.41, 0.43, 0.49)\} \end{aligned}$$

## Type-2 heptagonal fuzzy geometric aggregation operators

This section explores the geometric aggregation operator of a type-2 heptagonal fuzzy set.

### Type-2 heptagonal fuzzy weighted geometric aggregation operators

#### Definition 14

On a universe of discourse $\overset{\approx }{X}$, let us consider a set of *m* *T*2*HFNs*, $\overset{\approx }{\alpha }_{h1}, \overset{\approx }{\alpha }_{h2}, \overset{\approx }{\alpha }_{h3}, \ldots \overset{\approx }{\alpha }_{hm}$, the aggregation of the vectors $\overset{\approx }{\alpha }_{h1}, \overset{\approx }{\alpha }_{h2}, \overset{\approx }{\alpha }_{h3}, \ldots \overset{\approx }{\alpha }_{hm}$ is provided by the function *T*2*HFWG*, 

$$\begin{aligned} T2HFWG : \overset{\approx }{X}^{n} \mapsto \overset{\approx }{X} \end{aligned}$$

The operator is defined as,

$$\begin{aligned} T2HFWG\left(\overset{\approx }{\alpha }_{h1}, \overset{\approx }{\alpha }_{h2}, \overset{\approx }{\alpha }_{h3}, \ldots \overset{\approx }{\alpha }_{hm}\right)=\prod _{j=1}^{m}\left(\overset{\approx }{\alpha }_{h(j)}\right)^{\vec {\delta }_j} \end{aligned}$$

where, $\vec {\delta }=\left(\vec {\delta }_{1}, \vec {\delta }_{2}, \vec {\delta }_{3}, \ldots , \vec {\delta }_{m}\right)$ is the weight vector in which $\vec {\delta }_{j} \in [0,1]$ and $\sum _{j=1}^{m}|\vec {\delta }_{j}|=1$. Weights are numeral quantities and are utilized in this definition in general.

4 $$\begin{aligned} T2HFWG\left(\overset{\approx }{\alpha }_{h1}, \overset{\approx }{\alpha }_{h2}, \overset{\approx }{\alpha }_{h3}, \ldots \overset{\approx }{\alpha }_{hm}\right)=\prod _{j=1}^{m} \left[\left(\overset{\approx }{\alpha }_{hj}^p, \overset{\approx }{\alpha }_{hj}^s\right)\right]^{\vec {\delta }_{j}} \end{aligned}$$

If we consider the weight $\vec {\delta }=\frac{1}{n}$, then the *T*2*HFWG* is provided by,

5 $$\begin{aligned} T2HFWG\left(\overset{\approx }{\alpha }_{h1}, \overset{\approx }{\alpha }_{h2}, \overset{\approx }{\alpha }_{h3}, \ldots \overset{\approx }{\alpha }_{hm}\right)=\root n \of {\overset{\approx }{\alpha }_{h1}, \overset{\approx }{\alpha }_{h2}, \overset{\approx }{\alpha }_{h3}, \ldots \overset{\approx }{\alpha }_{hm}} \end{aligned}$$

#### Theorem 5

Consider the *m* $T2HFN's$ denoted by $\left(\overset{\approx }{\alpha }_{h1}, \overset{\approx }{\alpha }_{h2}, \overset{\approx }{\alpha }_{h3}, \ldots \overset{\approx }{\alpha }_{hm}\right);$ the value obtained after aggregating these *m* $T2HFN's$ by the *T*2*HFWG* operator is again a *T*2*HFN*

#### Proof

Consider, $|\overset{\approx }{\alpha }_{hj}|\le 1 \forall j=1, 2, \ldots , m$ and $\sum _{j=1}^{m} \vec {\delta }_{j}=1,$ it is obtained as follows,

$$\begin{aligned} \left|\left(\overset{\approx }{\alpha }_{h1}^{\vec {\delta _{1}}}. \overset{\approx }{\alpha }_{h2}^{\vec {\delta _{2}}}. \ldots . \overset{\approx }{\alpha }_{hm}^{\vec {\delta _{m}}}\right)\right| & = \left|\left(\overset{\approx }{\alpha }_{h1}^{\vec {\delta _{1}}}\right|. \left|\overset{\approx }{\alpha }_{h2}^{\vec {\delta _{2}}}\right|. \ldots . \left|\overset{\approx }{\alpha }_{hm}^{\vec {\delta _{m}}}\right)\right|\\ & \le 1^{\left(\vec {\delta }_{1}+\vec {\delta }_{2}+\ldots +\vec {\delta }_{m}\right)}\\ & =1 \end{aligned}$$

$$\square$$

#### Theorem 6

Consider $\left(\overset{\approx }{\alpha }_{h1}, \overset{\approx }{\alpha }_{h2}, \overset{\approx }{\alpha }_{c3}, \ldots \overset{\approx }{\alpha }_{cm}\right)$ to be *m* $T2HFN's$ and the weight values to be real values $\vec {\delta }=\vec {\delta }_{1}, \vec {\delta }_{2}, \ldots , \vec {\delta }_{m}$ and $|\vec {\delta }_{j}| \le 1$, then the following holds

1. (Idempotency) When $\overset{\approx }{\alpha }_{h1}=\overset{\approx }{\alpha }_{h2}=\overset{\approx }{\alpha }_{h3}= \ldots = \overset{\approx }{\alpha }_{hm}=\overset{\approx }{\alpha },$ then the operator becomes $T2HFWG\left(\overset{\approx }{\alpha }_{h1}, \overset{\approx }{\alpha }_{h2}, \overset{\approx }{\alpha }_{h3}, \ldots \overset{\approx }{\alpha }_{hm}\right)=\overset{\approx }{\alpha }$
2. (Boundedness) Consider $\textrm{max}_{j}|\overset{\approx }{\alpha }_{hj}|,$ then $\left|T2HFWG\left(\overset{\approx }{\alpha }_{h1}, \overset{\approx }{\alpha }_{h2}, \overset{\approx }{\alpha }_{h3}, \ldots \overset{\approx }{\alpha }_{hm}\right)\right| \le a$
3. (Monotonocity) Consider two sets of $T2HFWG \left\{ \overset{\approx }{\alpha }_{h1}, \overset{\approx }{\alpha }_{h2}, \overset{\approx }{\alpha }_{h3}, \ldots \overset{\approx }{\alpha }_{hm} \right\}$ and $\left\{ \overset{\approx }{\beta }_{h1}, \overset{\approx }{\beta }_{h2}, \overset{\approx }{\beta }_{h3}, \ldots \overset{\approx }{\beta }_{hm} \right\}$ such that $|\overset{\approx }{\alpha }_{h1}|\le |\overset{\approx }{\beta }_{h1}|, |\overset{\approx }{\alpha }_{h2}|\le |\overset{\approx }{\beta }_{h2}|, \ldots , |\overset{\approx }{\alpha }_{hm}|\le |\overset{\approx }{\beta }_{hm}|$, then the aggregation operator can be written as, $\left|T2HFWG\left(\overset{\approx }{\alpha }_{h1}, \overset{\approx }{\alpha }_{h2}, \overset{\approx }{\alpha }_{h3}, \ldots , \overset{\approx }{\alpha }_{hm}\right)\left| = \right|T2HFWG\left(\overset{\approx }{\beta }_{h1}, \overset{\approx }{\beta }_{h2}, \overset{\approx }{\beta }_{h3}, \ldots , \overset{\approx }{\beta }_{hm}\right)\right|$

#### Proof

By *T*2*HFWG* operator, consider

1. As $\overset{\approx }{\alpha }_{h1}=\overset{\approx }{\alpha }_{h2}= \ldots = \overset{\approx }{\alpha }_{hm} = \overset{\approx }{\alpha }$ and $\sum _{j=1}^{m} \vec {\delta }_{j}=1,$ then
  $$\begin{aligned} \left(\overset{\approx }{\alpha }_{h1}\right)^{\vec {\delta }_{1}}. \left(\overset{\approx }{\alpha }_{h2}\right)^{\vec {\delta }_{2}}. \ldots . \left(\overset{\approx }{\alpha }_{hm}\right)^{\vec {\delta }_{m}} & = \overset{\approx }{\alpha }^{\vec {\delta }_{1}+\vec {\delta }_{2}+\ldots +\vec {\delta }_{m}}\\ & = \overset{\approx }{\alpha } \end{aligned}$$
2. As $s=\textrm{max}_{j}|\overset{\approx }{\alpha }_{hj}|$ and $\vec {\delta }_{j} \in [0, 1] \forall j=1,2, \ldots m,$ then
  $$\begin{aligned} |(\overset{\approx }{\alpha }_{h1})^{\vec {\delta }_{1}}+ (\overset{\approx }{\alpha }_{h2})^{\vec {\delta }_{2}}+ \ldots + (\overset{\approx }{\alpha }_{hm})^{\vec {\delta }_{m}}| & = |\overset{\approx }{\alpha }_{h1}|^{\vec {\delta }_{1}} +|\overset{\approx }{\alpha }_{h2}|^{\vec {\delta }_{2}}+\ldots + |\overset{\approx }{\alpha }_{hm}|^ {\vec {\delta }_{m}}\\ & \le s^{\vec {\delta }_{1}}.s^{\vec {\delta }_{2}}.s^{\vec {\delta }_{3}}.\ldots .s^{\vec {\delta }_{m}}\\ & = s^{\left(\vec {\delta }_{1}+\vec {\delta }_{2}+\ldots +\vec {\delta }_{m}\right)}\\ & =s \end{aligned}$$
3. Here, $$|\overset{\approx }{\alpha }_{h1}| \le |\overset{\approx }{\beta }_{h1}|, |\overset{\approx }{\alpha }_{h2}| \le |\overset{\approx }{\beta }_{h2}|, \ldots , |\overset{\approx }{\alpha }_{hm}| \le |\overset{\approx }{\beta }_{hm}|,$$ hence
  $$\begin{aligned} |\overset{\approx }{\alpha }_{h1}^{\vec {\delta }_{1}}.\overset{\approx }{\alpha }_{h2}^{\vec {\delta }_{2}}.\ldots .\overset{\approx }{\alpha }_{hm}^{\vec {\delta }_{m}}| & = |\overset{\approx }{\alpha }_{h1}|^{\vec {\delta }_{1}}.|\overset{\approx }{\alpha }_{h2}|^{\vec {\delta }_{2}}.\ldots |\overset{\approx }{\alpha }_{hm}|^{\vec {\delta }_{m}}\\ & \le |\overset{\approx }{\beta }_{h1}|^{\vec {\delta }_{1}}.|\overset{\approx }{\beta }_{h2}|^{\vec {\delta }_{2}}.\ldots |\overset{\approx }{\beta }_{hm}|^{\vec {\delta }_{m}}\\ & = |T2HFWG(\overset{\approx }{\beta }_{h1}, \overset{\approx }{\beta }_{h2}, \ldots ,\overset{\approx }{\beta }_{hm})| \end{aligned}$$

$$\square$$

#### Example

Consider $\overset{\approx }{\alpha }_{h1}= \{(0.01, 0.12, 0.15, 0.18, 0.21, 0.25, 0.32), (0.22, 0.25, 0.29, 0.32, 0.37, 0.41, 0.43, 0.5)\}, \overset{\approx }{\alpha }_{h2}=\{(0.05, 0.14, 0.16, 0.22, 0.23, 0.27, 0.36), (0.20, 0.22, 0.25, 0.28, 0.30, 0.32, 0.35, 0.42)\}, \overset{\approx }{\alpha }_{h3}= \{(0.1, 0.15, 0.19, 0.24, 0.26, 0.31, 0.34), (0.15, 0.19, 0.29, 0.32, 0.37, 0.43, 0.45, 0.5)\}$ and $\overset{\approx }{\alpha }_{h4}= \{(0.12, 0.15, 0.19, 0.21, 0.27, 0.29, 0.32), (0.16, 0.25, 0.27, 0.32, 0.39, 0.41, 0.43, 0.5)\}$, having weights $\vec {\delta }_{1}=0.1, \vec {\delta }_{2}=0.25, \vec {\delta }_{3}=0.3, \vec {\delta }_{4}=0.35$, then the value can obtain by *T*2*HFWG* operator is, by Eq. 4

$$\begin{aligned} T2HFWG(\overset{\approx }{\alpha }_{h1}, \overset{\approx }{\alpha }_{h2}, \overset{\approx }{\alpha }_{h3}, \overset{\approx }{\alpha }_{h4}) & = \prod _{j=1}^{4} \left(\overset{\approx }{\alpha }_{j}\right)^{\delta _{j}}\\ & = \{(0.07, 0.14, 0.17, 0.22, 0.25, 0.28, 0.34),\\ & (0.18, 0.22, 0.27, 0.31, 0.35, 0.38, 0.41)\} \end{aligned}$$

### Type-2 heptagonal fuzzy ordered weighted geometric aggregation operators

#### Definition 15

On a universe of discourse $\overset{\approx }{X}$, let us consider a set of *m* $T2HFN's$, $\overset{\approx }{\alpha }_{h1}, \overset{\approx }{\alpha }_{h2}, \overset{\approx }{\alpha }_{h3}, \ldots \overset{\approx }{\alpha }_{hm}$, the aggregation of the vectors $\overset{\approx }{\alpha }_{h1}, \overset{\approx }{\alpha }_{h2}, \overset{\approx }{\alpha }_{h3}, \ldots \overset{\approx }{\alpha }_{hm}$ is provided by the function *T*2*HFOWA*, 

$$\begin{aligned} T2HFOWA : \overset{\approx }{X}^{n} \mapsto \overset{\approx }{X} \end{aligned}$$

The operator is defined as,

$$\begin{aligned} T2HFOWA(\overset{\approx }{\alpha }_{h1}, \overset{\approx }{\alpha }_{h2}, \overset{\approx }{\alpha }_{h3}, \ldots \overset{\approx }{\alpha }_{hm})=\sum \limits _{j=1}\left(\overset{\approx }{\alpha }_{ho(j)}\right)^{\vec {\delta }} \end{aligned}$$

Where $\vec {\delta }=\left(\vec {\delta }_{1}, \vec {\delta }_{2}, \vec {\delta }_{3}, \ldots , \vec {\delta }_{m}\right)$ is the weight vector in which $\vec {\delta }_{j} \in [0,1]$ and $\sum _{j=1}^{m}|\vec {\delta }_{j}|=1$ and $(o(1), o(2), o(3), \ldots , o(m))$ is the permutation of elements such that $|\overset{\approx }{\alpha }_{ohj}| \le |\overset{\approx }{\alpha }_{oh(j-1)}|$. Weights which are normal quantities utilized in this definition, so the definition is more general. The aggregation operator can also be written as,

$$\begin{aligned} T2HFOWG\left(\overset{\approx }{\alpha }_{h1}, \overset{\approx }{\alpha }_{h2}, \overset{\approx }{\alpha }_{h3}, \ldots \overset{\approx }{\alpha }_{hm}\right)=\sum \limits _{j=1}^{m}\left[\left(\overset{\approx }{\alpha }_{hj}^p, \overset{\approx }{\alpha }_{hj}^s\right)\right] \end{aligned}$$

If the weight consider as $\vec {\delta }=\frac{1}{n}$, then *T*2*HFOWG* is given by,

6 $$\begin{aligned} T2HFOWG\left(\overset{\approx }{\alpha }_{h1}, \overset{\approx }{\alpha }_{h2}, \overset{\approx }{\alpha }_{h3}, \ldots \overset{\approx }{\alpha }_{hm}\right)=\root n \of {\overset{\approx }{\alpha }_{h1}, \overset{\approx }{\alpha }_{h2}, \overset{\approx }{\alpha }_{h3}, \ldots \overset{\approx }{\alpha }_{hm}} \end{aligned}$$

#### Theorem 7

Consider the *m* $T2HFN's$ denoted by $\left(\overset{\approx }{\alpha }_{h1}, \overset{\approx }{\alpha }_{h2}, \overset{\approx }{\alpha }_{h3}, \ldots \overset{\approx }{\alpha }_{hm}\right);$ the value obtained after aggregating these *m* $T2HFN's$ by the *T*2*HFOWG* operator is again a *T*2*HFN*

#### Proof

This proof is consequent to 5, hence omitted. $\square$

#### Theorem 8

Consider $\left(\overset{\approx }{\alpha }_{h1}, \overset{\approx }{\alpha }_{h2}, \overset{\approx }{\alpha }_{h3}, \ldots \overset{\approx }{\alpha }_{hm}\right)$ to be *m* $T2HFN's$ and the weight values to be real values $\vec {\delta }=\vec {\delta }_{1}, \vec {\delta }_{2}, \ldots , \vec {\delta }_{m}$ and $|\vec {\delta }_{j}| \le 1$, then the following holds

1. (Idempotency) When $\overset{\approx }{\alpha }_{h1}=\overset{\approx }{\alpha }_{h2}=\overset{\approx }{\alpha }_{h3}= \ldots = \overset{\approx }{\alpha }_{hm}=\overset{\approx }{\alpha },$ then the operator becomes $T2HFOWG\left(\overset{\approx }{\alpha }_{h1}, \overset{\approx }{\alpha }_{h2}, \overset{\approx }{\alpha }_{h3}, \ldots \overset{\approx }{\alpha }_{hm}\right)=\overset{\approx }{\alpha }$
2. (Boundedness) Consider $\textrm{max}_{j}|\overset{\approx }{\alpha }_{hj}|,$ then $|T2HFOWG\left(\overset{\approx }{\alpha }_{h1}, \overset{\approx }{\alpha }_{h2}, \overset{\approx }{\alpha }_{h3}, \ldots \overset{\approx }{\alpha }_{hm}\right)| \le a$
3. (Monotonocity) Consider two sets of $T2HFOWG \left\{ \overset{\approx }{\alpha }_{h1}, \overset{\approx }{\alpha }_{h2}, \overset{\approx }{\alpha }_{h3}, \ldots \overset{\approx }{\alpha }_{hm} \right\}$ and $\left\{ \overset{\approx }{\beta }_{h1}, \overset{\approx }{\beta }_{h2}, \overset{\approx }{\beta }_{h3}, \ldots \overset{\approx }{\beta }_{hm} \right\}$ such that $|\overset{\approx }{\alpha }_{h1}|\le |\overset{\approx }{\beta }_{h1}|, |\overset{\approx }{\alpha }_{h2}|\le |\overset{\approx }{\beta }_{h2}|, \ldots , |\overset{\approx }{\alpha }_{hm}|\le |\overset{\approx }{\beta }_{hm}|$, then the aggregation operator can be written as, $\left|T2HFOWG\left(\overset{\approx }{\alpha }_{h1}, \overset{\approx }{\alpha }_{h2}, \overset{\approx }{\alpha }_{h3}, \ldots , \overset{\approx }{\alpha }_{hm}\right)| = |T2HFOWG\left(\overset{\approx }{\beta }_{h1}, \overset{\approx }{\beta }_{h2}, \overset{\approx }{\beta }_{h3}, \ldots , \overset{\approx }{\beta }_{hm}\right)\right|$

#### Proof

This proof is consequent to 6, hence omitted. $\square$

#### Example

Consider $\overset{\approx }{\alpha }_{h1}= \{(0.01, 0.12, 0.15, 0.18, 0.21, 0.25, 0.32), (0.22, 0.25, 0.29, 0.32, 0.37, 0.41, 0.43, 0.5)\}, \overset{\approx }{\alpha }_{h2}=\{(0.05, 0.14, 0.16, 0.22, 0.23, 0.27, 0.36), (0.20, 0.22, 0.25, 0.28, 0.30, 0.32, 0.35, 0.42)\}, \overset{\approx }{\alpha }_{h3}= \{(0.1, 0.15, 0.19, 0.24, 0.26, 0.31, 0.34), (0.15, 0.19, 0.29, 0.32, 0.37, 0.43, 0.45, 0.5)\}$ and $\overset{\approx }{\alpha }_{h4}= \{(0.12, 0.15, 0.19, 0.21, 0.27, 0.29, 0.32), (0.16, 0.25, 0.27, 0.32, 0.39, 0.41, 0.43, 0.5)\}$, having weights $\vec {\delta }_{1}=0.1, \vec {\delta }_{2}=0.25, \vec {\delta }_{3}=0.3, \vec {\delta }_{4}=0.35$, then the value can obtain by *T*2*HFOWG* operator is, by Eq. 6

$$\begin{aligned} T2HFWG\left(\overset{\approx }{\alpha }_{h1}, \overset{\approx }{\alpha }_{h2}, \overset{\approx }{\alpha }_{h3}, \overset{\approx }{\alpha }_{h4}\right) & = \prod _{j=1}^{4} \left(\overset{\approx }{\alpha }_{o(j)}\right)^{\delta _{j}}\\ & = \left(\overset{\approx }{\alpha }_{h3}\right)^{0.35}.\left(\overset{\approx }{\alpha }_{h4}\right)^{0.3}.\left(\overset{\approx }{\alpha }_{h1}\right)^{0.25}. \left(\overset{\approx }{\alpha }_{h2}\right)^{0.1}\\ & = \{(0.05, 0.14, 0.18, 0.21, 0.25, 0.28, 0.33),\\ & (0.17,0.22,0.28,0.32,0.37,0.41,0.43,0.49)\} \end{aligned}$$

## Type-2 heptagonal fuzzy power arithmetic operator

The definition and properties of type-2 heptagonal fuzzy power weighted arithmetic operators are explained in this section.

### Type-2 heptagonal fuzzy power weighted arithmetic operator

#### Definition 16

On a universe of discourse $\overset{\approx }{X}$, let us consider a set of *m* *T*2*HFNs*, $\overset{\approx }{\alpha }_{h1}, \overset{\approx }{\alpha }_{h2}, \overset{\approx }{\alpha }_{h3}, \ldots \overset{\approx }{\alpha }_{hm}$, the aggregation of the vectors $\overset{\approx }{\alpha }_{h1}, \overset{\approx }{\alpha }_{h2}, \overset{\approx }{\alpha }_{h3}, \ldots \overset{\approx }{\alpha }_{hm}$ is provided by the function *T*2*HFWP*, 

$$\begin{aligned} T2HFWP : \overset{\approx }{X}^{n} \mapsto \overset{\approx }{X} \end{aligned}$$

The operator is defined as,

$$\begin{aligned} T2HFOWA\left(\overset{\approx }{\alpha }_{h1}, \overset{\approx }{\alpha }_{h2}, \overset{\approx }{\alpha }_{h3}, \ldots \overset{\approx }{\alpha }_{hm}\right)=\sum \limits _{j=1}^{m}\left(\vec {\delta }\overset{\approx }{\alpha }_{h(j)}^{g}\right)^\frac{1}{g} \end{aligned}$$

Where $\vec {\delta }=\left(\vec {\delta }_{1}, \vec {\delta }_{2}, \vec {\delta }_{3}, \ldots , \vec {\delta }_{m}\right)$ is the weight vector in which $\vec {\delta }_{j} \in [0,1]$ and parameter $g \in (0,\infty )$, $\sum _{j=1}^{m}|\vec {\delta }_{j}|=1$. Weights which are normal quantities utilized in this definition, so the definition is more general. The aggregation operator can also be written as,

7 $$\begin{aligned} T2HFPWA\left(\overset{\approx }{\alpha }_{h1}, \overset{\approx }{\alpha }_{h2}, \overset{\approx }{\alpha }_{h3}, \ldots \overset{\approx }{\alpha }_{hm}\right)=\left(\sum \limits _{j=1}^{m} \delta _{j}[\overset{\approx }{\alpha }_{hj}^{p}, \overset{\approx }{\alpha }_{hj}^{s}]^{g}\right)^{\frac{1}{g}} \end{aligned}$$

The *T*2*HFWP* operator can be made into an arithmetic average operator when considering the weight as $\vec {\delta }=1$, which is written as,

8 $$\begin{aligned} T2HFWP\left(\overset{\approx }{\alpha }_{h1}, \overset{\approx }{\alpha }_{h2}, \overset{\approx }{\alpha }_{h3}, \ldots \overset{\approx }{\alpha }_{hm}\right)=\left(\sum \limits _{j=1}^{m} \frac{1}{n} \overset{\approx }{\alpha }_{hj}^{g}\right)^{\frac{1}{g}} \end{aligned}$$

#### Theorem 9

Consider *m* $T2HFN's$ denoted by $\overset{\approx }{\alpha }_{h1}, \overset{\approx }{\alpha }_{h2}, \overset{\approx }{\alpha }_{h3}, \ldots \overset{\approx }{\alpha }_{hm}$. The value obtained after aggregating these *m* *T*2*HFNs* by the *T*2*HFPWA* operator is again a *T*2*HFN*.

#### Proof

When $|\overset{\approx }{\alpha }_{hj}| \le 1 \forall j=1, 2, \ldots , m$ and $g \ge 0,$ hence, $|\overset{\approx }{\alpha }|_{hj}^g \le 1$ also $\sum _{j=1}^{m} \vec {\delta }_{j}=1$

$$\begin{aligned} |\vec {\delta }_{1}.\overset{\approx }{\alpha }_{h1}^{g}+\vec {\delta }_{2}.\overset{\approx }{\alpha }_{h2}^{g}+\ldots +\vec {\delta }_{m}.\overset{\approx }{\alpha }_{hm}^{g}| & \le \vec {\delta }_{1}|\overset{\approx }{\alpha }_{h1}^{g}|+\vec {\delta }_{2}.|\overset{\approx }{\alpha }_{h2}^{g}|+\ldots +\vec {\delta }_{m}.|\overset{\approx }{\alpha }_{hm}^{g}|\\ & \vec {\delta }_{1}+\vec {\delta }_{2}+\ldots +\vec {\delta }_{m}\\ & = 1 \end{aligned}$$

It is clear that $\left|T2HFWPA\left(\overset{\approx }{\alpha }_{h1}, \overset{\approx }{\alpha }_{h2}, \ldots , \overset{\approx }{\alpha }_{hm}\right)\right| \le 1$ is also a *T*2*HFN*. $\square$

#### Theorem 10

Consider $\left(\overset{\approx }{\alpha }_{h1}, \overset{\approx }{\alpha }_{h2}, \overset{\approx }{\alpha }_{h3}, \ldots \overset{\approx }{\alpha }_{hm}\right)$ to be *m* $T2HFN's$ and the weight values to be real values $\vec {\delta }=\vec {\delta }_{1}, \vec {\delta }_{2}, \ldots , \vec {\delta }_{m}$ and $|\vec {\delta }_{j}| \le 1$, then the following holds

1. (Idempotency) When $\overset{\approx }{\alpha }_{h1}=\overset{\approx }{\alpha }_{h2}=\overset{\approx }{\alpha }_{h3}= \ldots = \overset{\approx }{\alpha }_{hm}=\overset{\approx }{\alpha },$ then the operator becomes $T2HFPWA\left(\overset{\approx }{\alpha }_{h1}, \overset{\approx }{\alpha }_{h2}, \overset{\approx }{\alpha }_{h3}, \ldots \overset{\approx }{\alpha }_{hm}\right)=\overset{\approx }{\alpha }$
2. (Boundedness) Consider $a=\textrm{max}_{j}|\overset{\approx }{\alpha }_{hj}|,$ then $|T2HFPWA\left(\overset{\approx }{\alpha }_{h1}, \overset{\approx }{\alpha }_{h2}, \overset{\approx }{\alpha }_{h3}, \ldots \overset{\approx }{\alpha }_{hm}\right)| \le a$
3. (Monotonocity) Consider two sets of $T2HFPWA \left\{ \overset{\approx }{\alpha }_{h1}, \overset{\approx }{\alpha }_{h2}, \overset{\approx }{\alpha }_{h3}, \ldots \overset{\approx }{\alpha }_{hm} \right\}$ and $\left\{ \overset{\approx }{\beta }_{h1}, \overset{\approx }{\beta }_{h2}, \overset{\approx }{\beta }_{h3}, \ldots \overset{\approx }{\beta }_{hm} \right\}$ such that $|\overset{\approx }{\alpha }_{h1}|\le |\overset{\approx }{\beta }_{h1}|, |\overset{\approx }{\alpha }_{h2}|\le |\overset{\approx }{\beta }_{h2}|, \ldots , |\overset{\approx }{\alpha }_{hm}|\le |\overset{\approx }{\beta }_{hm}|$, then the aggregation operator can be written as, $\left|T2HFPWA\left(\overset{\approx }{\alpha }_{h1}, \overset{\approx }{\alpha }_{h2}, \overset{\approx }{\alpha }_{h3}, \ldots , \overset{\approx }{\alpha }_{hm}\right)\right| = \left|T2HFPWA\left(\overset{\approx }{\beta }_{h1}, \overset{\approx }{\beta }_{h2}, \overset{\approx }{\beta }_{h3}, \ldots , \overset{\approx }{\beta }_{hm}\right)\right|$

#### Proof

By *T*2*HFPWA*

1. As $\overset{\approx }{\alpha }_{h1}=\overset{\approx }{\alpha }_{h2}=\ldots =\overset{\approx }{\alpha }_{hm}=\overset{\approx }{\alpha }$ and $\vec {\delta }_{j}=1,$ hence
  $$\begin{aligned} \left(\left(\vec {\delta }_{1}.\overset{\approx }{\alpha }_{h1}^{g}+\vec {\delta }_{2}.\overset{\approx }{\alpha }_{h2}^{g}+\dots +\vec {\delta }_{m}.\overset{\approx }{\alpha }_{hm}^{g}\right)^{\frac{1}{g}} \right.& \left.=\left(\vec {\delta }_{1}+\vec {\delta }_{2}+\dots +\vec {\delta }_{m}\right).\overset{\approx }{\alpha }^{g}\right)^{\frac{1}{g}}\\ & = \overset{\approx }{\alpha } \end{aligned}$$
2. As $a=\textrm{max}_{j}|\overset{\approx }{\alpha }_{hj}|$ and $\vec {\delta }_{j} \in [0, 1]$ for every $j=1, 2, \ldots , m$
  $$\begin{aligned} |\vec {\delta }_{1}. \overset{\approx }{\alpha }_{h1}^{g}+\vec {\delta }_{2}. \overset{\approx }{\alpha }_{h2}^{g}+\ldots +\vec {\delta }_{m}. \overset{\approx }{\alpha }_{hm}^{g}| & = \vec {\delta }_{1}. |\overset{\approx }{\alpha }_{h1}|^{g}+\vec {\delta }_{2}. |\overset{\approx }{\alpha }_{h2}^{g}|+\ldots +\vec {\delta }_{m}. |\overset{\approx }{\alpha }_{hm}^{g}|\\ & = \vec {\delta }_{1}. a^{g}+\vec {\delta }_{2}. a^{g}+\ldots +\vec {\delta }_{m}. a^{g}\\ & =\left(\vec {\delta }_{1}+\vec {\delta }_{2}+\ldots +\vec {\delta }_{m}\right).a^{g}\\ & = a^{g} \end{aligned}$$
  Hence, $$|\vec {\delta }_{1}. \overset{\approx }{\alpha }_{h1}^{g}+\vec {\delta }_{2}. \overset{\approx }{\alpha }_{h2}^{g}+\ldots +\vec {\delta }_{m}. \overset{\approx }{\alpha }_{hm}^{g}|^{\frac{1}{g}} \le (a^g)^{\frac{1}{g}}=g$$
3. Here consider, $|\overset{\approx }{\alpha }_{h1}| \le |\overset{\approx }{\beta }_{h1}|, |\overset{\approx }{\alpha }_{h2}| \le |\overset{\approx }{\beta }_{h2}|, \ldots , |\overset{\approx }{\alpha }_{hm}| \le |\overset{\approx }{\beta }_{hm}|$ thus $|\overset{\approx }{\alpha }_{h1}|^{g} \le |\overset{\approx }{\beta }_{h1}|^{g}, |\overset{\approx }{\alpha }_{h2}|^{g} \le |\overset{\approx }{\beta }_{h2}|^{g}, \ldots , |\overset{\approx }{\alpha }_{hm}|^{g} \le |\overset{\approx }{\beta }_{hm}|^{g}$ as $g>0$
  $$\begin{aligned} |\vec {\delta }_{1}. \overset{\approx }{\alpha }_{h1}^{g}+\vec {\delta }_{2}. \overset{\approx }{\alpha }_{h2}^{g}+\ldots +\vec {\delta }_{m}. \overset{\approx }{\alpha }_{hm}^{g}| & \le \vec {\delta }_{1}. |\overset{\approx }{\alpha }_{h1}|^{g}+\vec {\delta }_{2}. |\overset{\approx }{\alpha }_{h2}^{g}|+\ldots +\vec {\delta }_{m}. |\overset{\approx }{\alpha }_{hm}^{g}|\\ & \le \vec {\delta }_{1}. |\overset{\approx }{\beta }_{h1}|^{g}+\vec {\delta }_{2}. |\overset{\approx }{\beta }_{h2}^{g}|+\ldots +\vec {\delta }_{m}. |\overset{\approx }{\beta }_{hm}^{g}|\\ & \le |T2HFPW(\overset{\approx }{\beta }_{h1}, \overset{\approx }{\beta }_{h2}, \ldots , \overset{\approx }{\beta }_{hm} )| \end{aligned}$$

$$\square$$

#### Example

Consider $\overset{\approx }{\alpha }_{h1}= \{(0.01, 0.12, 0.15, 0.18, 0.21, 0.25, 0.32), (0.22, 0.25, 0.29, 0.32, 0.37, 0.41, 0.43, 0.5)\}, \overset{\approx }{\alpha }_{h2}=\{(0.05, 0.14, 0.16, 0.22, 0.23, 0.27, 0.36), (0.20, 0.22, 0.25, 0.28, 0.30, 0.32, 0.35, 0.42)\}, \overset{\approx }{\alpha }_{h3}= \{(0.1, 0.15, 0.19, 0.24, 0.26, 0.31, 0.34), (0.15, 0.19, 0.29, 0.32, 0.37, 0.43, 0.45, 0.5)\}$ and $\overset{\approx }{\alpha }_{h4}= \{(0.12, 0.15, 0.19, 0.21, 0.27, 0.29, 0.32), (0.16, 0.25, 0.27, 0.32, 0.39, 0.41, 0.43, 0.5)\}$, having weights $\vec {\delta }_{1}=0.1, \vec {\delta }_{2}=0.25, \vec {\delta }_{3}=0.3, \vec {\delta }_{4}=0.35$, then the value can obtain by *T*2*HFWG* operator is, by Eq. 8

$$\begin{aligned} T2HFPWA(\overset{\approx }{\alpha }_{h1}, \overset{\approx }{\alpha }_{h2}, \overset{\approx }{\alpha }_{h3}, \overset{\approx }{\alpha }_{h4}) & = \left(\sum \limits _{j=1}^{4} \delta _{j}(\overset{\approx }{\alpha }_{j})^2\right)^{\frac{1}{2}}\\ & = \{(0.08, 0.14, 0.18, 0.22, 0.25, 0.29, 0.34),\\ & (0.18, 0.22, 0.27, 0.31, 0.35, 0.39, 0.41)\} \end{aligned}$$

**Fig. 6:**
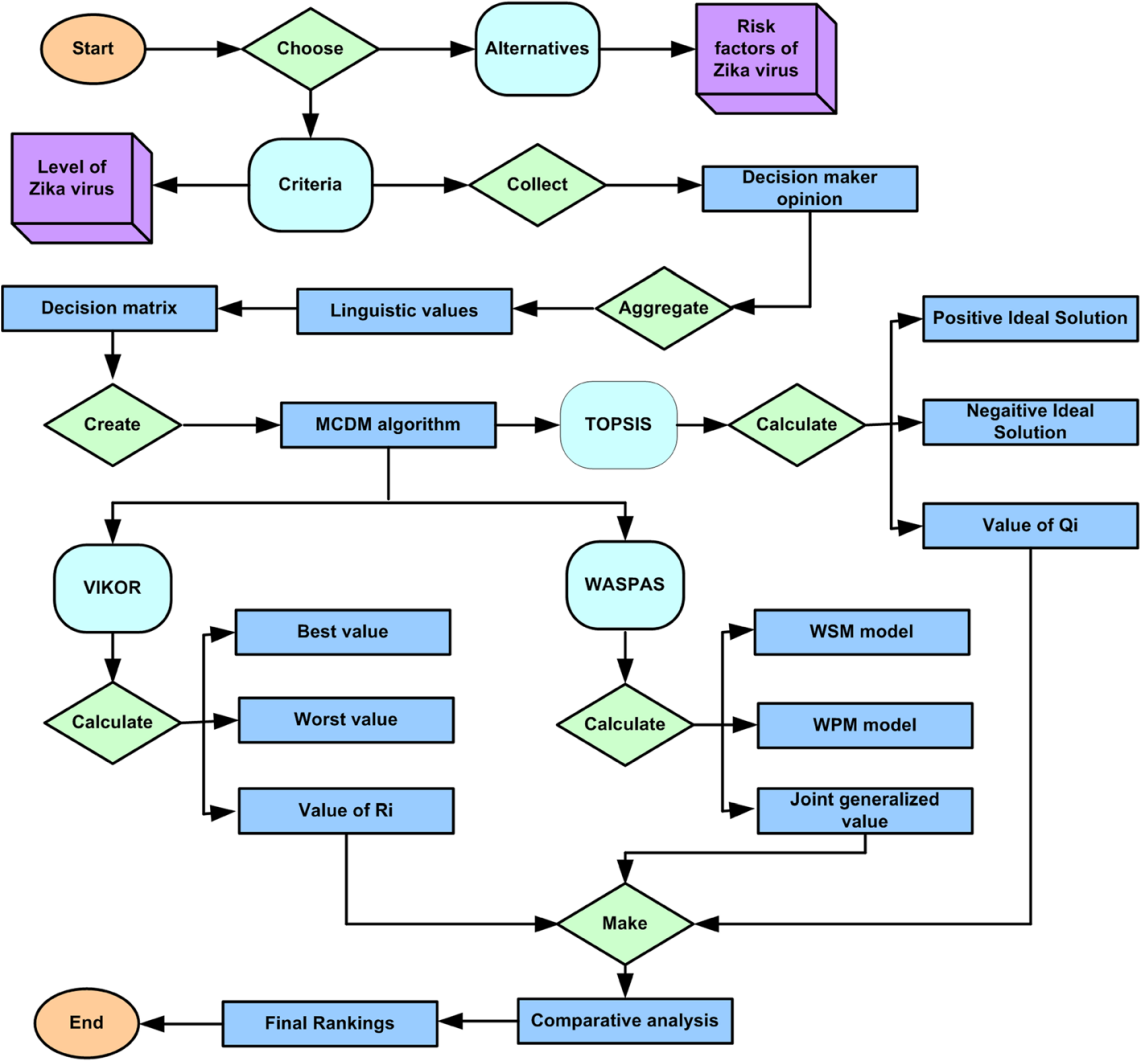
Flowchart of the proposed work

### Type-2 heptagonal fuzzy power ordered weighted arithmetic operator

#### Definition 17

On a universe of discourse $\overset{\approx }{X}$, let us consider a set of *m* *T*2*HFNs*, $\overset{\approx }{\alpha }_{h1}, \overset{\approx }{\alpha }_{h2}, \overset{\approx }{\alpha }_{h3}, \ldots \overset{\approx }{\alpha }_{hm}$, the aggregation of the vectors $\overset{\approx }{\alpha }_{h1}, \overset{\approx }{\alpha }_{h2}, \overset{\approx }{\alpha }_{h3}, \ldots \overset{\approx }{\alpha }_{hm}$ is provided by the function *T*2*HFPOWA*, 

$$\begin{aligned} T2HFPOWA : \overset{\approx }{X}^{n} \mapsto \overset{\approx }{X} \end{aligned}$$

The operator is defined as,

$$\begin{aligned} T2HFOWA\left(\overset{\approx }{\alpha }_{h1}, \overset{\approx }{\alpha }_{h2}, \overset{\approx }{\alpha }_{h3}, \ldots \overset{\approx }{\alpha }_{hm}\right)=\sum \limits _{j=1}^{m}\left(\vec {\delta }\overset{\approx }{\alpha }_{h(j)}^{g}\right)^\frac{1}{g} \end{aligned}$$

Where $\vec {\delta }=\left(\vec {\delta }_{1}, \vec {\delta }_{2}, \vec {\delta }_{3}, \ldots , \vec {\delta }_{m}\right)$ is the weight vector in which $\vec {\delta }_{j} \in [0,1]$ and parameter $g \in (0,\infty )$, $\sum _{j=1}^{m}|\vec {\delta }_{j}|=1$. Weights are normal quantities utilized in this definition, so the definition is more general. The aggregation operator can also be written as,

9 $$\begin{aligned} T2HFPOWA(\overset{\approx }{\alpha }_{h1}, \overset{\approx }{\alpha }_{h2}, \overset{\approx }{\alpha }_{h3}, \ldots \overset{\approx }{\alpha }_{hm})=\left(\sum \limits _{j=1}^{m} \delta _{j}\left[(\overset{\approx }{\alpha }_{hj}^p,\overset{\approx }{\alpha }_{hj}^s) \right]^{g}\right)^{\frac{1}{g}} \end{aligned}$$

The *T*2*HFOWP* operator can be made into an arithmetic average operator when considering the weight as $\vec {\delta }=1$, which is written as,

10 $$\begin{aligned} T2HFOWP(\overset{\approx }{\alpha }_{h1}, \overset{\approx }{\alpha }_{h2}, \overset{\approx }{\alpha }_{h3}, \ldots \overset{\approx }{\alpha }_{hm})=\left(\sum \limits _{j=1}^{m} \frac{1}{n} \overset{\approx }{\alpha }_{ho(j)}^{g}\right)^{\frac{1}{g}} \end{aligned}$$

#### Theorem 11

Consider *m* $T2HFN's$ denoted by $\overset{\approx }{\alpha }_{h1}, \overset{\approx }{\alpha }_{h2}, \overset{\approx }{\alpha }_{h3}, \ldots \overset{\approx }{\alpha }_{hm}$. The value obtained after aggregating these *m* $T2HFN's$ by the *T*2*HFPWG* operator is again a *T*2*HFN*.

#### Proof

This proof is consequent to Theorem 9, hence omitted. $\square$

#### Theorem 12

Consider $\left(\overset{\approx }{\alpha }_{h1}, \overset{\approx }{\alpha }_{h2}, \overset{\approx }{\alpha }_{h3}, \ldots \overset{\approx }{\alpha }_{cm}\right)$ to be *m* $T2HFN's$ and the weight values to be real values $\vec {\delta }=\vec {\delta }_{1}, \vec {\delta }_{2}, \ldots , \vec {\delta }_{m}$ and $|\vec {\delta }_{j}| \le 1$, then the following holds

1. (Idempotency) When $\overset{\approx }{\alpha }_{h1}=\overset{\approx }{\alpha }_{h2}=\overset{\approx }{\alpha }_{h3}= \ldots = \overset{\approx }{\alpha }_{hm}=\overset{\approx }{\alpha },$ then the operator becomes $T2HFPWG\left(\overset{\approx }{\alpha }_{h1}, \overset{\approx }{\alpha }_{h2}, \overset{\approx }{\alpha }_{h3}, \ldots \overset{\approx }{\alpha }_{hm}\right)=\overset{\approx }{\alpha }$
2. (Boundedness) Consider $a=\textrm{max}_{j}|\overset{\approx }{\alpha }_{hj}|,$ then $|T2HFPWG(\overset{\approx }{\alpha }_{h1}, \overset{\approx }{\alpha }_{h2}, \overset{\approx }{\alpha }_{h3}, \ldots \overset{\approx }{\alpha }_{hm})| \le a$
3. (Monotonocity) Consider two sets of $T2HFPWG \left\{ \overset{\approx }{\alpha }_{h1}, \overset{\approx }{\alpha }_{h2}, \overset{\approx }{\alpha }_{h3}, \ldots \overset{\approx }{\alpha }_{hm} \right\}$ and $\left\{ \overset{\approx }{\beta }_{h1}, \overset{\approx }{\beta }_{h2}, \overset{\approx }{\beta }_{h3}, \ldots \overset{\approx }{\beta }_{hm} \right\}$ such that $|\overset{\approx }{\alpha }_{h1}|\le |\overset{\approx }{\beta }_{h1}|, |\overset{\approx }{\alpha }_{h2}|\le |\overset{\approx }{\beta }_{h2}|, \ldots , |\overset{\approx }{\alpha }_{hm}|\le |\overset{\approx }{\beta }_{hm}|$, then the aggregation operator can be written as, $|T2HFPWG\left(\overset{\approx }{\alpha }_{h1}, \overset{\approx }{\alpha }_{h2}, \overset{\approx }{\alpha }_{h3}, \ldots , \overset{\approx }{\alpha }_{hm}\right)| = |T2HFPWG\left(\overset{\approx }{\beta }_{h1}, \overset{\approx }{\beta }_{h2}, \overset{\approx }{\beta }_{h3}, \ldots , \overset{\approx }{\beta }_{hm}\right)|$

#### Proof

This proof is consequent to Theorem 10, hence omitted. $\square$

#### Example

Consider $\overset{\approx }{\alpha }_{h1}= \{(0.01, 0.12, 0.15, 0.18, 0.21, 0.25, 0.32), (0.22, 0.25, 0.29, 0.32, 0.37, 0.41, 0.43, 0.5)\}, \overset{\approx }{\alpha }_{h2}=\{(0.05, 0.14, 0.16, 0.22, 0.23, 0.27, 0.36), (0.20, 0.22, 0.25, 0.28, 0.30, 0.32, 0.35, 0.42)\}, \overset{\approx }{\alpha }_{h3}= \{(0.1, 0.15, 0.19, 0.24, 0.26, 0.31, 0.34), (0.15, 0.19, 0.29, 0.32, 0.37, 0.43, 0.45, 0.5)\}$ and $\overset{\approx }{\alpha }_{h4}= \{(0.12, 0.15, 0.19, 0.21, 0.27, 0.29, 0.32), (0.16, 0.25, 0.27, 0.32, 0.39, 0.41, 0.43, 0.5)\}$, having weights $\vec {\delta }_{1}=0.1, \vec {\delta }_{2}=0.25, \vec {\delta }_{3}=0.3, \vec {\delta }_{4}=0.35$, then the value can obtain by *CT*2*FPWOA* operator is, by Eq. 10

$$\begin{aligned} T2HFPWG(\overset{\approx }{\alpha }_{h1}, \overset{\approx }{\alpha }_{h2}, \overset{\approx }{\alpha }_{h3}, \overset{\approx }{\alpha }_{h4}) & = \left(\prod _{j=1}^{4} \delta _{o(j)}\left(\overset{\approx }{\alpha }_{o(j)}\right)^2\right)^{\frac{1}{2}}\\ & = \{(0.089, 0.142, 0.178, 0.215, 0.248, 0.285, 0.331), \\ & (0.178, 0.228, 0.280, 0.316, 0.369, 0.409, 0.429)\} \end{aligned}$$

## Type-2 heptagonal fuzzy numbers extended to TOPSIS, VIKOR and WASPAS

This section explains type-2 fuzzy heptagonal numbers with the distance-based MCDM methods such as TOPSIS and WASPAS and outranking and distance-based method VIKOR.

### Case study-Zika viral disease

The Zika virus was first identified in a rhesus monkey in the Zika Forest of Uganda during a yellow fever research project conducted by the Rockefeller Foundation in 1947. The first human cases of Zika virus were detected in Uganda and the United Republic of Tanzania. In the 1950’s Zika virus was detected sporadically in Africa and Asia, with cases reported in countries such as Nigeria, India, Malaysia, and Indonesia. However, the virus remained relatively obscure and was not associated with major outbreaks. The first significant outbreak of Zika occurred in 2007 on the Pacific island of Yap in Micronesia. This outbreak showed that Zika could cause large-scale epidemics and provided valuable information about its clinical features, which included rash, conjunctivitis, and joint pain. In between 2013 and 2014, Zika virus outbreaks occurred in several Pacific islands, including French Polynesia, Easter Island, the Cook Islands, and New Caledonia. These outbreaks were notable for their association with neurological complications, including GBS. The Zika virus began to spread rapidly in the Americas. In May 2015, Brazil reported its first cases of Zika virus infection. The virus quickly spread throughout South and Central America and the Caribbean. This period saw an alarming rise in cases of microcephaly and other congenital abnormalities in newborns, leading to the declaration of a Public Health Emergency of International Concern by the WHO in February 2016.

The link between Zika virus infection during pregnancy and severe birth defects, including microcephaly, was confirmed through epidemiological and laboratory studies. Research also confirmed that Zika could be transmitted sexually and through blood transfusions, expanding the known routes of transmission. The incidence of Zika virus infection has decreased significantly since the peak of the epidemic in 2015–2016. However, sporadic cases and outbreaks continue to occur in various regions. Research into vaccines, antiviral treatments, and improved diagnostic methods is ongoing. Public health efforts focus on mosquito control, surveillance, and education to prevent future outbreaks. Recent research continues to explore the long-term effects of Zika virus infection, especially in children born with congenital Zika syndrome. Vaccine development has progressed, with several candidates entering clinical trials. The WHO and other health organizations continue to monitor and respond to Zika virus activity worldwide.

For pregnant women, the Zika virus poses significant risks due to its potential to cause severe birth defects and other complications. This work explains the most valuable risk factors of the Zika virus.

1. **Travel to Zika-Endemic Areas:** Pregnant women traveling to regions with active Zika transmission such as parts of South America, Central America, the Caribbean, and Southeast Asia are at higher risk of contracting the virus. Traveling to Zika-endemic areas poses significant risks for pregnant women due to the potential for Zika virus infection, which can severely affect both the mother and the developing fetus [54]. If a pregnant woman contracts the Zika virus, there is a risk of vertical transmission from the mother to the fetus through the placenta [55]. Infection during pregnancy can lead to congenital Zika syndrome, which includes severe fetal brain defects such as microcephaly, brain abnormalities, and other serious developmental issues [56].
2. **Living in Zika-Endemic Areas:** Residing in areas where the Zika virus is prevalent increases the risk of mosquito bites from infected Aedes mosquitoes. Living in Zika-endemic areas significantly increases the risk for pregnant women of contracting the Zika virus through mosquito bites, leading to potential vertical transmission to the fetus [57]. This can result in severe fetal brain defects like microcephaly and other developmental issues known as congenital Zika syndrome. Pregnant women may also face higher risks of miscarriage, stillbirth, and preterm birth. Consistent use of mosquito repellent, protective clothing, and staying in screened and air-conditioned environments are crucial preventive measures [58].
3. **Unprotected Sexual Activity:** Engaging in unprotected sex with a partner who has traveled to or resides in an area with Zika transmission can lead to sexual transmission of the virus [59]. Unprotected sexual activity is a significant risk factor for Zika virus transmission. The virus can be transmitted sexually from an infected partner, even if the partner does not exhibit symptoms [60]. This mode of transmission can occur before, during, and after the appearance of symptoms. For pregnant women, this is particularly concerning as it increases the risk of the virus reaching the developing fetus, leading to severe outcomes like congenital Zika syndrome. Symptoms of Zika in adults include fever, rash, joint pain, and red eyes, but many cases are asymptomatic [61].
4. **Lack of Mosquito Precautions:** Lack of mosquito precautions significantly increases the risk of Zika virus transmission [62]. Without protective measures, individuals are more susceptible to bites from Aedes mosquitoes, the primary carriers of the Zika virus. This increases the likelihood of infection, particularly in areas where these mosquitoes are prevalent [63]. Pregnant women are at heightened risk as the virus can be transmitted to the fetus, causing severe birth defects like microcephaly and congenital Zika syndrome. Failure to use mosquito repellents, wear protective clothing, and ensure living spaces are mosquito-free exacerbates the spread. Standing water, where mosquitoes breed, further heightens the risk if not managed properly. Public health efforts emphasize the importance of these precautions to curb the spread and protect vulnerable populations [64].
5. **Blood Transfusions:** Receiving blood transfusions in areas with Zika transmission can pose a risk, although this is less common compared to mosquito bites and sexual transmission. Blood transmission increases the risk of Zika virus for pregnant women through several pathways [65]. If a pregnant woman receives a blood transfusion or organ transplant from an infected donor, she can contract the virus [66]. Additionally, sharing needles or other blood-contaminated instruments can lead to infection. Once infected, the virus can cross the placental barrier and infect the fetus, leading to severe consequences such as congenital Zika syndrome, which includes microcephaly and other neurological abnormalities [67]. Routine blood screening for Zika virus in endemic areas is crucial to prevent such transmissions. Pregnant women should also avoid situations where blood transmission is possible and ensure that all medical procedures involving blood are handled safely and hygienically. These precautions are vital to protect both the mother and the developing fetus from the serious risks associated with Zika virus infection [68].
6. **Previous Infection with Flaviviruses:** Previous infection with other flaviviruses, such as dengue, and West Nile virus, can potentially complicate the spread and impact of Zika virus in several ways [69]. These flaviviruses are closely related and share similarities in their genetic makeup, which can lead to cross-reactive immune responses in individuals who have been previously infected [70]. This cross-reactivity means that antibodies produced in response to one flavivirus infection may also recognize and interact with the Zika virus, albeit to varying degrees of effectiveness [71]. In some cases, antibodies generated from a previous flavivirus infection can enhance the ability of the Zika virus to enter cells and replicate. This phenomenon, known as antibody-dependent enhancement, may result in more severe Zika virus infections upon subsequent exposure [72].
7. **Poor Prenatal Care:** Lack of access to regular prenatal care can prevent early detection and management of Zika-related complications in pregnancy [73]. Poor prenatal care can contribute to the spread of the Zika virus by limiting opportunities for early detection, monitoring, and management of infections among pregnant women [74]. Inadequate prenatal care may delay or prevent women from receiving essential information about Zika virus prevention, symptoms, and testing. This can result in missed opportunities for timely screening and diagnosis of Zika virus infections during pregnancy [75]. Additionally, insufficient prenatal care may lead to missed opportunities for monitoring fetal development and early signs of congenital Zika syndrome.
8. **Immunocompromised State:** Pregnant women with weakened immune systems may have a higher susceptibility to infections, including the Zika virus, and may experience more severe symptoms and complications [76]. An immunocompromised state increases susceptibility to Zika virus infection due to weakened immune defenses, which may impair the body’s ability to effectively respond to and clear the virus. Individuals with conditions such as HIV/AIDS, cancer undergoing chemotherapy, organ transplant recipients on immunosuppressive therapy, and those with certain autoimmune disorders are particularly vulnerable [77]. Immunocompromised individuals may experience prolonged and more severe Zika virus symptoms, as their immune systems struggle to mount an adequate response. This can lead to higher viral loads in the bloodstream and potentially increase the duration and likelihood of transmitting the virus to others via bodily fluids [78]. It is crucial for healthcare providers to closely monitor and manage Zika virus infections in immunocompromised patients to minimize complications and prevent further transmission within healthcare settings and communities.
9. **Close contact with infected individuals:** Close contact with infected individuals can increase the risk of Zika virus transmission to pregnant women through direct exposure to bodily fluids containing the virus [79]. Close contact with infected individuals, particularly those exhibiting symptoms and who have recently traveled to Zika-endemic areas, increases the likelihood of exposure. Pregnant women are at heightened risk because the virus can cross the placental barrier, potentially causing severe birth defects like microcephaly and other developmental abnormalities in the fetus [80].
10. **Inadequate Housing Conditions:** Inadequate housing conditions contribute to Zika virus transmission among pregnant women by creating environments conducive to mosquito breeding and increased human-mosquito contact. Poorly constructed and overcrowded housing often lacks proper sanitation and drainage, leading to stagnant water where Aedes mosquitoes breed. This increases the likelihood of mosquito bites and subsequent Zika virus transmission [81]. Homes without screens on windows and doors fail to protect against mosquitoes, while lack of air conditioning may compel residents to keep windows open, further exposing them to mosquito bites indoors. Additionally, limited access to mosquito repellents, protective clothing, and bed nets in inadequate housing exacerbates the risk [82]. Poor housing conditions are also associated with reduced access to healthcare services, hindering timely diagnosis, prevention, and management of Zika virus infections during pregnancy. Addressing these housing challenges through improved infrastructure, mosquito control efforts, community education, and enhanced healthcare access is essential to protect pregnant women and their unborn children from Zika virus-related risks.

### Criteria evaluation

In this section, we describe the process of evaluating the criteria associated with the risk factors for Zika virus transmission. A thorough assessment was conducted to identify and prioritize the most significant risk factors, utilizing expert evaluations and MCDM methods. The criteria were selected based on their relevance to the Zika virus transmission dynamics and their potential impact on public health interventions.

1. **Incubation Period (**$C_1$**)** The incubation period refers to the time between exposure to the Zika virus and the development of symptoms. During this phase, individuals may be asymptomatic but still infectious, contributing to silent transmission, particularly through mosquito bites and sexual contact [3]. The incubation period is typically 3–14 days.
2. **Acute Phase (**$C_2$**)** The acute phase is when the infected individual experiences symptoms such as fever, rash, conjunctivitis, and arthralgia. This phase is critical for identifying cases and mitigating further spread, as individuals may transmit the virus to others through mosquito bites, sexual transmission, or blood transfusion [83].
3. **Post-Acute Phase (**$C_3$**)** The post-acute phase involves the prolonged presence of the virus in bodily fluids, such as semen, urine, and saliva, even after symptoms resolve. This phase is particularly important for sexual transmission and persistence in communities [84].
4. **Congenital Infection (**$C_4$**)** Maternal Zika virus infection during pregnancy can result in congenital Zika syndrome, leading to severe fetal complications like microcephaly, neurological deficits, and developmental delays. This phase is critical for understanding vertical transmission risks [85].
5. **Long-Term Monitoring and Care (**$C_5$**)** Zika virus infections, especially congenital and neurological complications, require long-term monitoring and care. Children with congenital Zika syndrome need continuous medical, developmental, and psychosocial support. Adults may experience Guillain-Barre Syndrome, necessitating long-term rehabilitation [86].

The evaluation process provided a clear ranking of the key risk factors, which will guide targeted interventions for reducing Zika virus transmission.

### Calculation

**Fig. 7:**
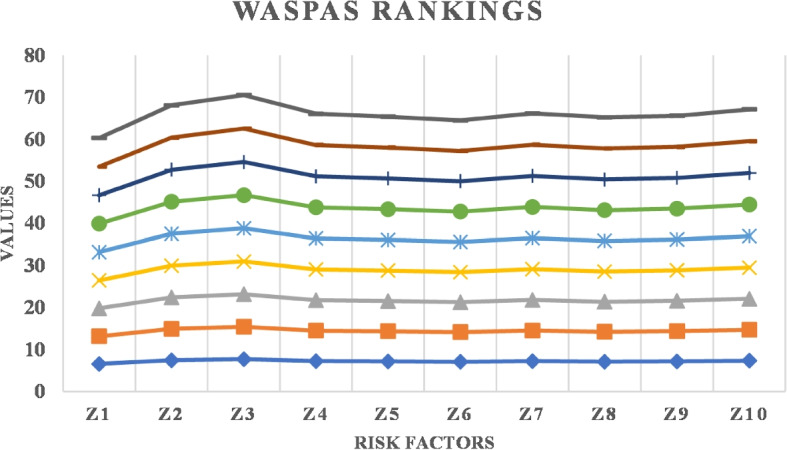
WASPAS rankings

By the Flowchart (Fig. 6), the steps explained at Algorithm 1, 2, 3 and 4. Table 2 explicates the risk factors and their notation and Table 3 shows the notation and criteria of the proposed work. Here, the weights were determined using a fuzzy weighting approach, ensuring a balanced representation of expert opinions and criteria importance. Tables 4, 5 and 6 providing the opinions of three decision-makers about the risk factors of the Zika virus. The proposed new score values of T2HFN expounds in Table 7. Final rankings of TOPSIS, VIKOR and WASPAS rankings are shown in the Tables 8, 9 and Fig. 7. Through this, the dangerous risk factors of the Zika virus are calculated and arranged in an order.

**Table 2:** Risk factors and notations

Notation	Alternatives
$Z_1$	Travel to Zika-Endemic areas
$Z_2$	Living in Zika-Endemic Areas
$Z_3$	Unprotected Sexual Activity
$Z_4$	Lack of Mosquito Precautions
$Z_5$	Immunocompromised State
$Z_6$	Previous Exposure to Zika Virus
$Z_7$	Poor Prenatal Care
$Z_8$	Blood Transfusions
$Z_9$	Close Contact with Infected Individuals
$Z_{10}$	Inadequate Housing Conditions

**Table 3:** Criteria and notations

Notation	Criteria
$C_1$	Incubation Period
$C_2$	Acute Phase
$C_3$	Post-Acute Phase
$C_4$	Congenital Infection (Fetal Stage)
$C_5$	Long-Term Monitoring and Care

**Table 4:** First decision maker opinion

Alternatives	$C_1$	$C_2$	$C_3$	$C_4$	$C_5$
$Z_1$	VL	L	ML	MH	VH
$Z_2$	L	M	H	H	H
$Z_3$	L	M	MH	VH	EH
$Z_4$	L	L	MH	H	VH
$Z_5$	L	M	ML	MH	VH
$Z_6$	L	L	ML	M	MH
$Z_7$	L	M	ML	M	H
$Z_8$	L	M	M	VH	EH
$Z_9$	L	M	M	M	VH
$Z_{10}$	L	M	MH	H	H

**Table 5:** Second decision maker opinion

Alternatives	$C_1$	$C_2$	$C_3$	$C_4$	$C_5$
$Z_1$	VL	L	ML	M	H
$Z_2$	VL	L	MH	MH	VH
$Z_3$	VL	M	ML	VH	EH
$Z_4$	VL	ML	M	H	H
$Z_5$	L	ML	MH	H	VH
$Z_6$	L	M	MH	MH	H
$Z_7$	L	MH	M	H	VH
$Z_8$	VL	ML	ML	MH	VH
$Z_9$	VL	MH	M	H	VH
$Z_{10}$	VL	ML	M	H	VH

**Table 6:** Third decision maker opinion

Alternatives	$C_1$	$C_2$	$C_3$	$C_4$	$C_5$
$Z_1$	L	L	MH	H	VH
$Z_2$	L	ML	MH	H	H
$Z_3$	L	ML	M	H	EH
$Z_4$	L	ML	MH	MH	H
$Z_5$	VL	L	H	MH	H
$Z_6$	VL	ML	MH	H	VH
$Z_7$	VL	ML	M	H	VH
$Z_8$	VL	L	ML	H	EH
$Z_9$	VL	ML	M	MH	MH
$Z_{10}$	VL	ML	MH	MH	H

The calculation of the entire matrix is not feasible due to certain limitations. Therefore, the calculation for the risk factor $Z_1$ is demonstrated below. The average value of three decision-makers is,

$$\begin{aligned} A_{11} & = \frac{(VL+VL+L)}{3} \\ & = \{(0.033, 0.133, 0.233, 0.333, 0.433, 0.533, 0.633), (0.083, 0.183, 0.283, 0.383, 0.483, 0.583, 0.683)\}\\ Sc(A_{11}) & = (0.083+0.183+0.283+0.383+0.483+0.583+0.133+0.233+0.333+0.433+0.533+0.633)\\ & -\frac{1}{7}(0.05+0.05+0.05+0.05+0.05+0.05+0.2+0.05+0.05+0.05+0.05+0.05+0.05+0.2)\\ & = 4.3- 0.1428\\ Sc(A_{11}) & = 4.157 \end{aligned}$$

Following this step, as per the TOPSIS method, distance-based measures were calculated. Additionally, the compromise solution using the VIKOR method was determined, along with the computation of WSM and WPM values. For $Z_1$, the TOPSIS values are calculated as,

$$\begin{aligned} d_1^+ & = \sqrt{(4.157 - 4.557)^2 + (4.957 - 7.343)^2 + (6.943 - 8.857)^2 + (8.471 - 10.088)^2 + (9.813 - 11.156)^2} \\ & = \sqrt{(0.400)^2 + (2.386)^2 + (1.914)^2 + (1.617)^2 + (1.343)^2} \\ & = \sqrt{0.160 + 5.698 + 3.665 + 2.615 + 1.804} \\ & = \sqrt{13.942} = 3.734 \\ \\ d_1^- & = \sqrt{(4.157 - 4.157)^2 + (4.957 - 6.157)^2 + (6.943 - 6.557)^2 + (8.471 - 8.471)^2 + (9.813 - 9.473)^2} \\ & = \sqrt{(0.000)^2 + (1.200)^2 + (0.386)^2 + (0.000)^2 + (0.340)^2} \\ & = \sqrt{0.000 + 1.440 + 0.149 + 0.000 + 0.116} \\ & = \sqrt{1.705} = 0.515 \\ r_1 & = \frac{d_1^-}{d_1^+ + d_1^-} = \frac{0.515}{3.734 + 0.515} = \frac{0.515}{4.249} = 0.121 \end{aligned}$$

**Table 7:** Score values of the type-2 heptagonal fuzzy numbers

Alternatives	$C_1$	$C_2$	$C_3$	$C_4$	$C_5$
$Z_1$	4.157	4.957	6.943	8.471	9.813
$Z_2$	4.557	6.157	8.857	9.198	9.813
$Z_3$	4.557	6.957	7.343	10.088	11.156
$Z_4$	4.557	5.757	8.130	9.198	9.813
$Z_5$	4.557	6.157	7.343	8.857	10.088
$Z_6$	4.557	6.157	7.730	8.471	9.473
$Z_7$	4.557	7.343	6.557	8.857	10.088
$Z_8$	4.157	6.157	6.557	9.473	10.892
$Z_9$	4.157	7.343	7.343	8.471	9.813
$Z_{10}$	4.157	6.557	8.130	9.198	10.088

**Table 8:** TOPSIS rankings

Alternatives	$d_{i}^{+}$	$d_{i}^{-}$	$r_{i}$	Rankings
$Z_1$	3.734	0.515	0.121	10
$Z_2$	2.001	2.745	0.578	2
$Z_3$	1.562	3.198	0.672	1
$Z_4$	2.376	1.979	0.454	6
$Z_5$	2.521	1.657	0.396	8
$Z_6$	2.851	1.725	0.376	9
$Z_7$	2.819	2.526	0.472	5
$Z_8$	2.703	2.111	0.438	7
$Z_9$	2.621	2.535	0.491	4
$Z_{10}$	1.801	2.437	0.575	3

**Table 9:** VIKOR rankings

Alternatives	$S_{i}$	$R_{i}$	$Q_{i}$	Rankings
$Z_1$	0.934	1.037	1.000	10
$Z_2$	0.373	0.798	0.319	2
$Z_3$	0.165	0.658	0.002	1
$Z_4$	0.471	0.798	0.383	4
$Z_5$	0.514	0.761	0.363	3
$Z_6$	0.601	1.725	0.736	7
$Z_7$	0.479	1.000	0.656	5
$Z_8$	0.611	1.000	0.741	8
$Z_9$	0.691	1.000	0.794	9
$Z_{10}$	0.569	1.000	0.713	6

Compromise solution of VIKOR is calculated as,

$$\begin{aligned} Q_{i} & = (0.5)\left[\frac{S_{i}-S^{*}}{S^{-}- S^{*}}\right] + (1-0.5) \left[\frac{R_{i}-R^{*}}{R^{-} - R^{*}}\right]\\ Q_{1} & = (0.5) \left[\frac{0.934-0.165}{0.934-0.165}\right]+(0.5)\left[\frac{1.037-0.658}{1.725-0.658}\right]\\ Q_{1} & = 1.000 \end{aligned}$$

Continue the process for all alternatives. Sorting $Q_{i}$ in ascending order to determine the rankings. The rankings are $Z_3<Z_2<Z_5<Z_4<Z_7<Z_{10}<Z_6<Z_8<Z_9<Z_1$. By this $Z_3$ got the first risk factor which is the cause of the Zika virus.

**Table 10:** Final FIR rankings

Alternatives	TOPSIS	VIKOR	WASPAS	FIR
$Z_1$	10	10	10	10
$Z_2$	2	2	2	2
$Z_3$	1	1	1	1
$Z_4$	6	4	5	6
$Z_5$	8	3	7	8
$Z_6$	9	7	8	9
$Z_7$	5	5	4	5
$Z_8$	7	8	9	7
$Z_9$	4	9	6	4
$Z_{10}$	3	6	3	3

**Fig. 8:**
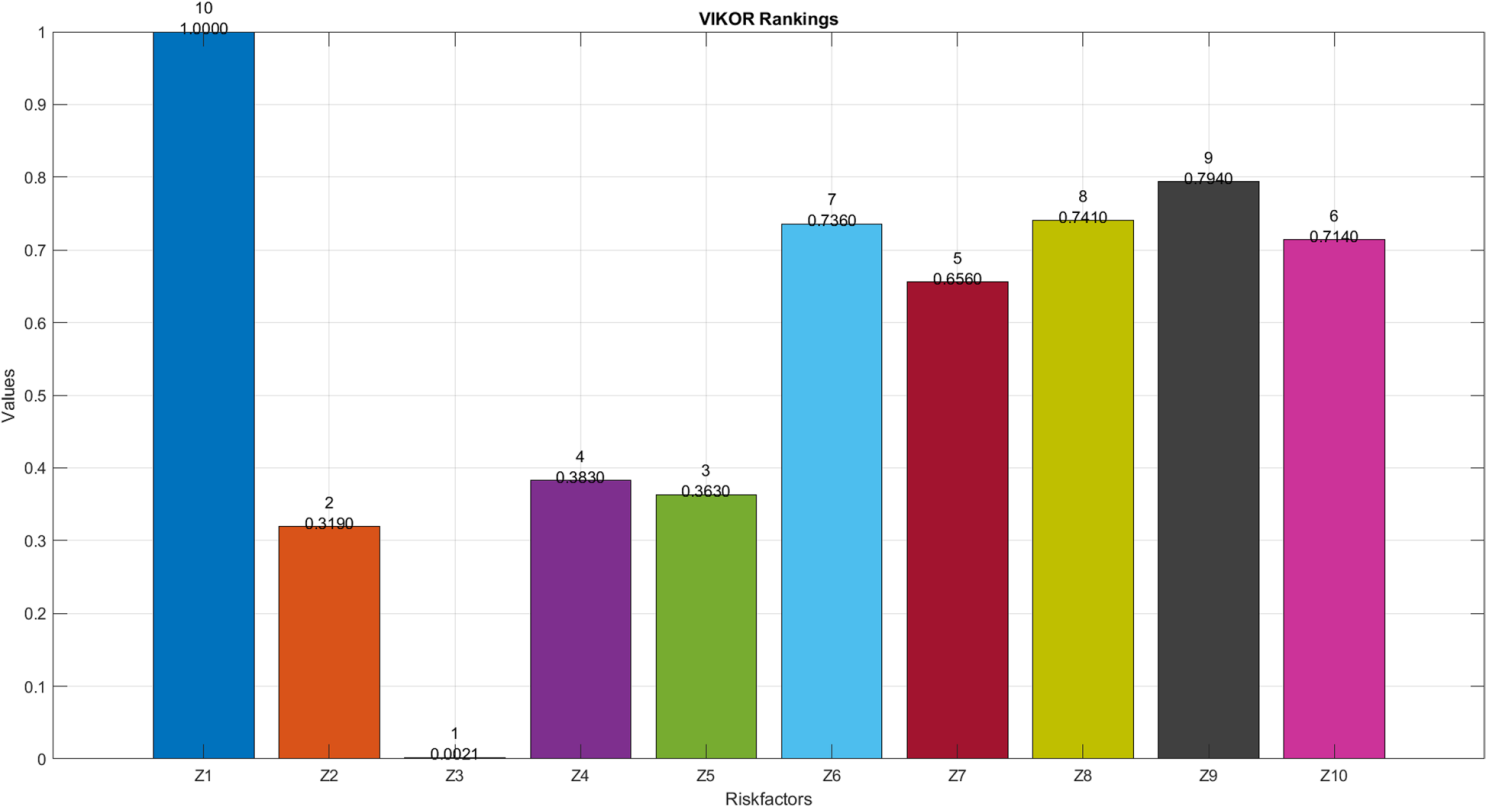
VIKOR rankings

**Fig. 9:**
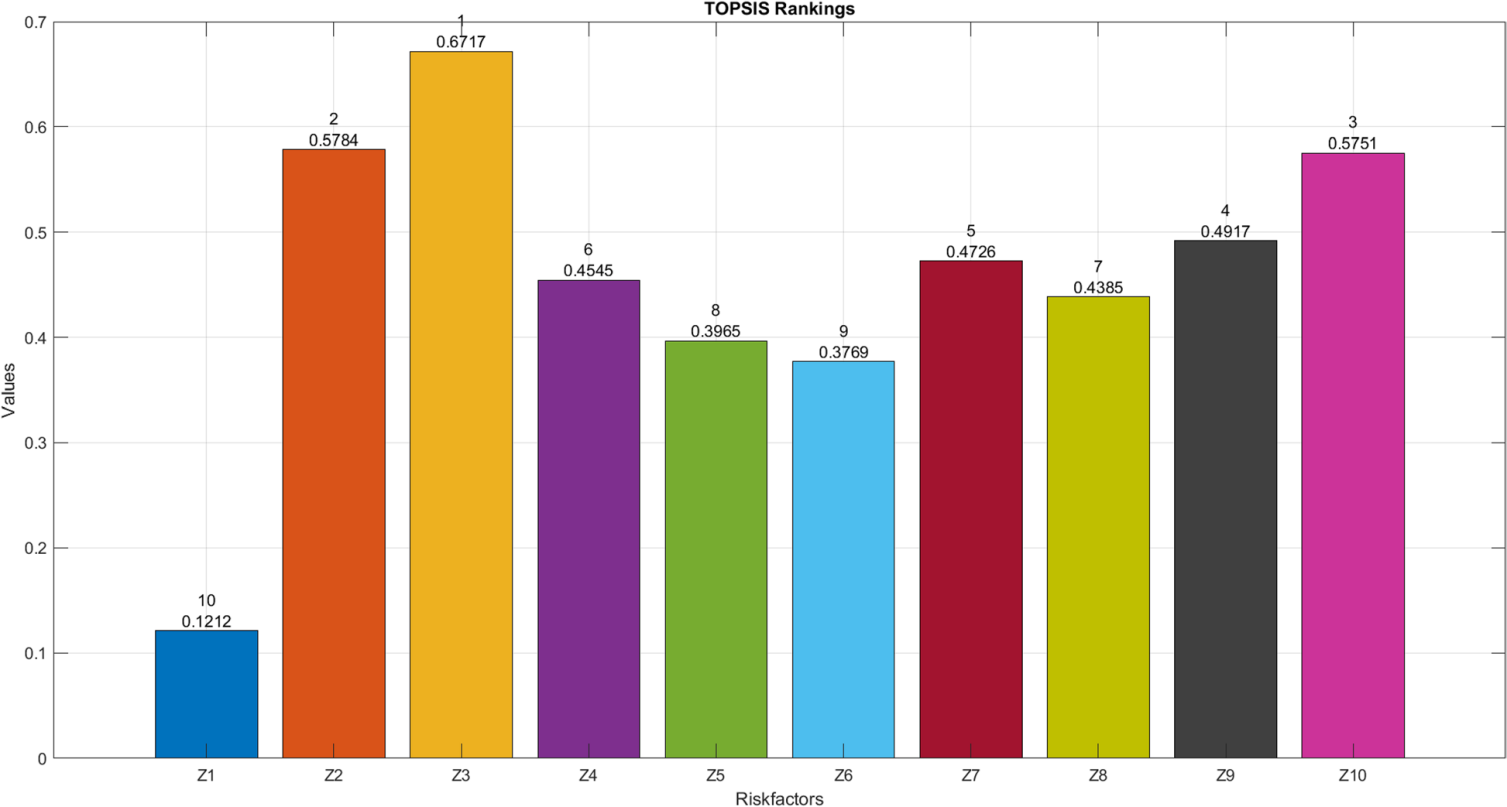
TOPSIS rankings

**Figure:**
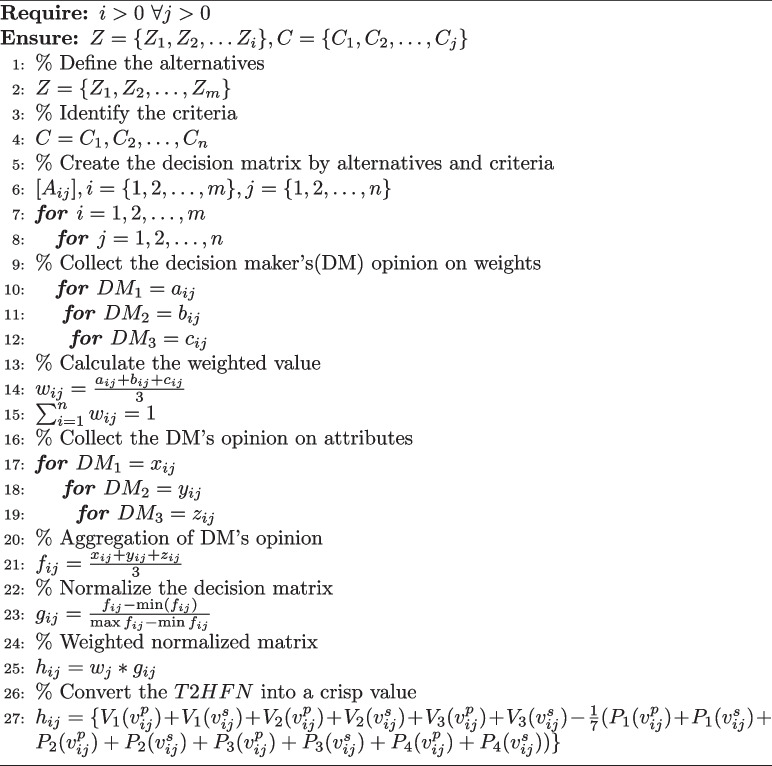
**Algorithm 1** Choose the best risk factor from $Z=Z_{1}, Z_{2}, \ldots , Z_{10}$ **(Standard procedures in MADM)**

**Figure:**
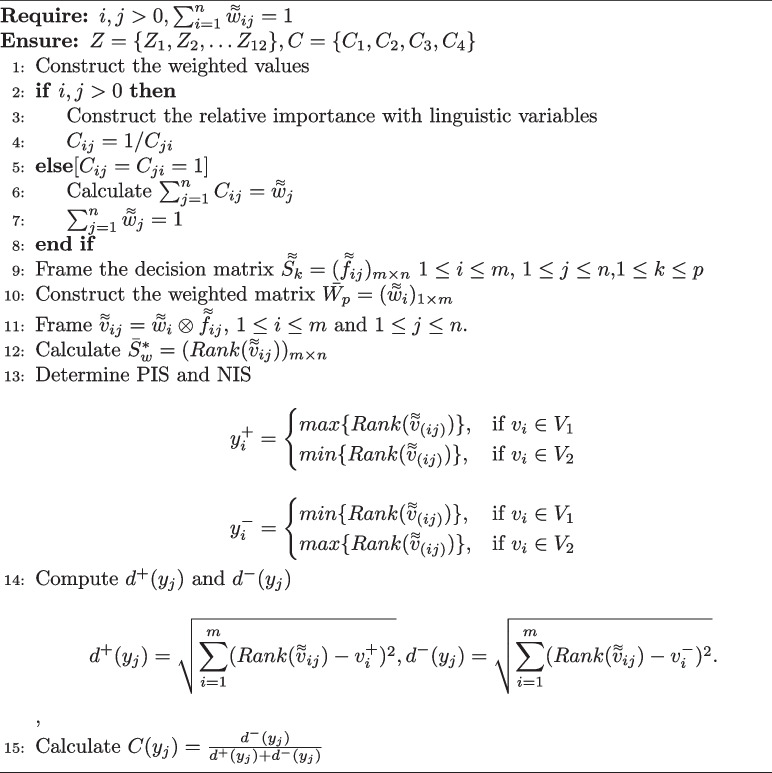
**Algorithm 2** Choose the best risk factor from $Z = Z_{1}, Z_{2}, \ldots Z_{12} {\textbf {(TOPSIS)}}$

**Figure:**
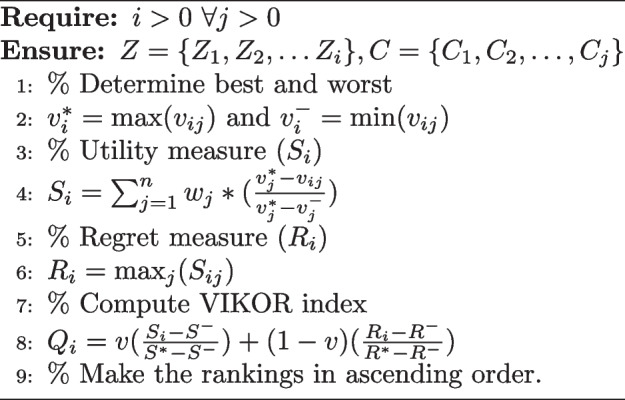
**Algorithm 3** Choose the best risk factor from $Z=Z_{1}, Z_{2}, \ldots , Z_{12}$ **(VIKOR)**

**Figure:**
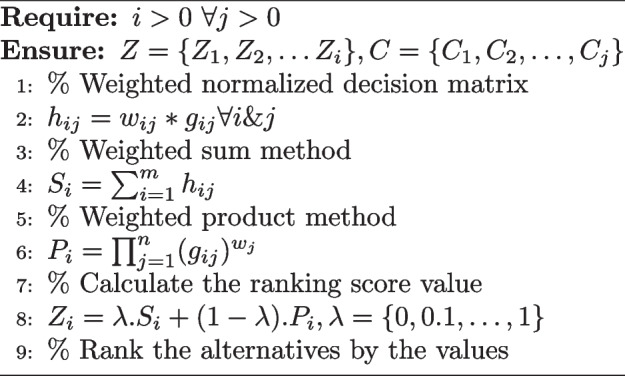
**Algorithm 4** Choose the best risk factor from $Z=Z_{1}, Z_{2}, \ldots , Z_{10}$ **(WASPAS)**

## Discussion

The application of Type-2 heptagonal fuzzy sets with multiple operators in MCDM for identifying risk factors of the Zika virus represents a significant advancement in handling uncertainty and imprecision in complex decision environments. This discussion section explores the implications, effectiveness, and potential limitations of the proposed approach.

**Fig. 10:**
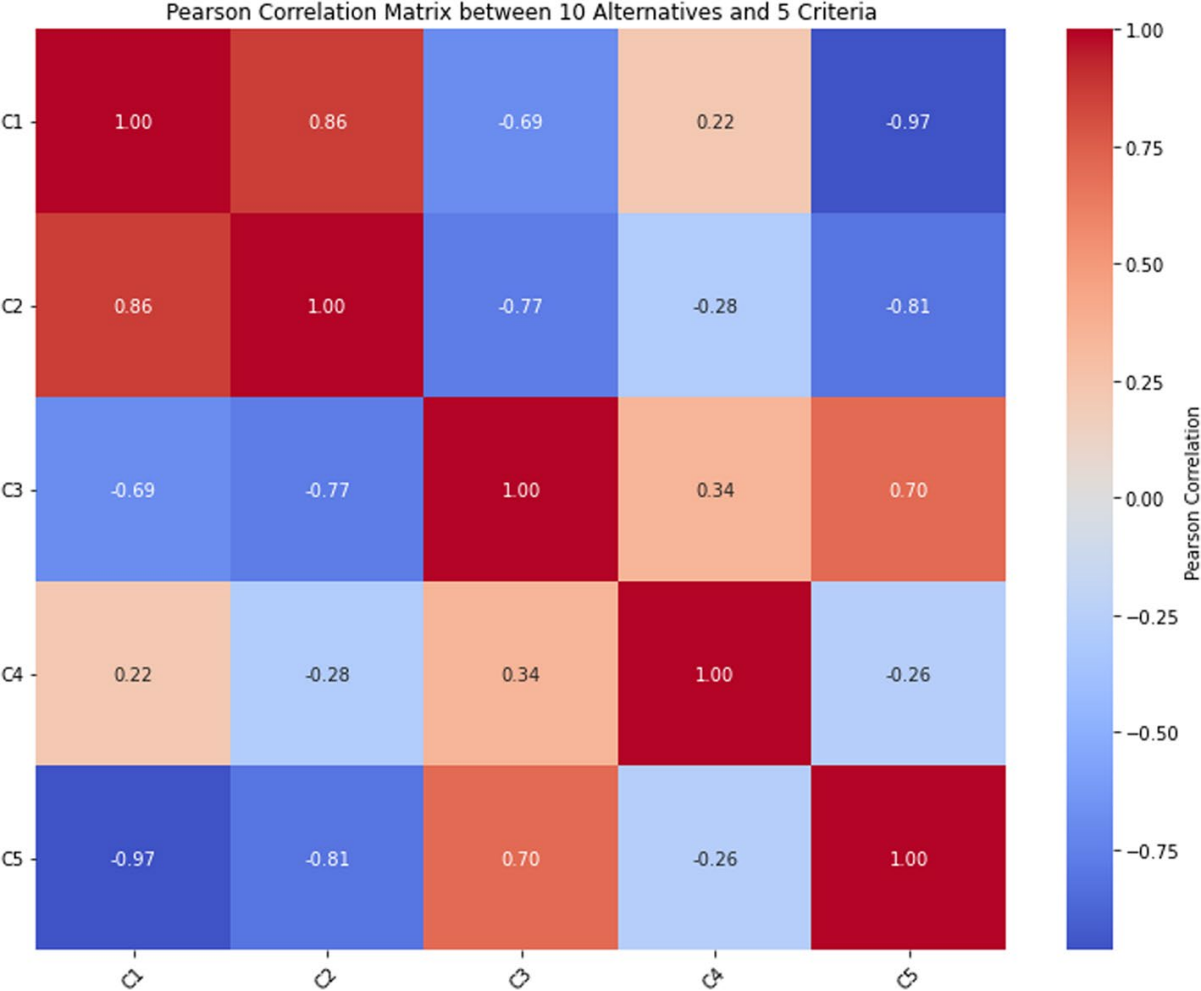
Pearson correlation co-efficient values in between alternatives criteria

In the proposed work, the rankings of risk factors of the Zika virus are explained with the situations of patients as criteria. The risk factor $Z_{3}$ unprotected sexual activity got the first place in the system of risk factors. $Z_{8}$ blood transfusions are in the second place of the dangerous risk factor. Continuously $Z_{10}, Z_{9}$ and $Z_{7}$ are in the next positions. The distance-based methods such as TOPSIS and WASPAS provide the rankings in ascending order. The outranking and distance-based method VIKOR provides the ranking in descending order. Figures 7, 8 and 9 are shown and explicated the rankings. When the different methods provide different rankings, then the fuzzy inference rankings (FIR) method can be utilized to make the final rankings of risk factors. By the if and then rules the FIR provided the rankings and it is shown in Table 10. By Table 10 the risk factor $Z_{3}$ unprotected sex is making the high-risk factor for the Zika Virus spreading increasing the seriousness of the patients.

**Fig. 11:**
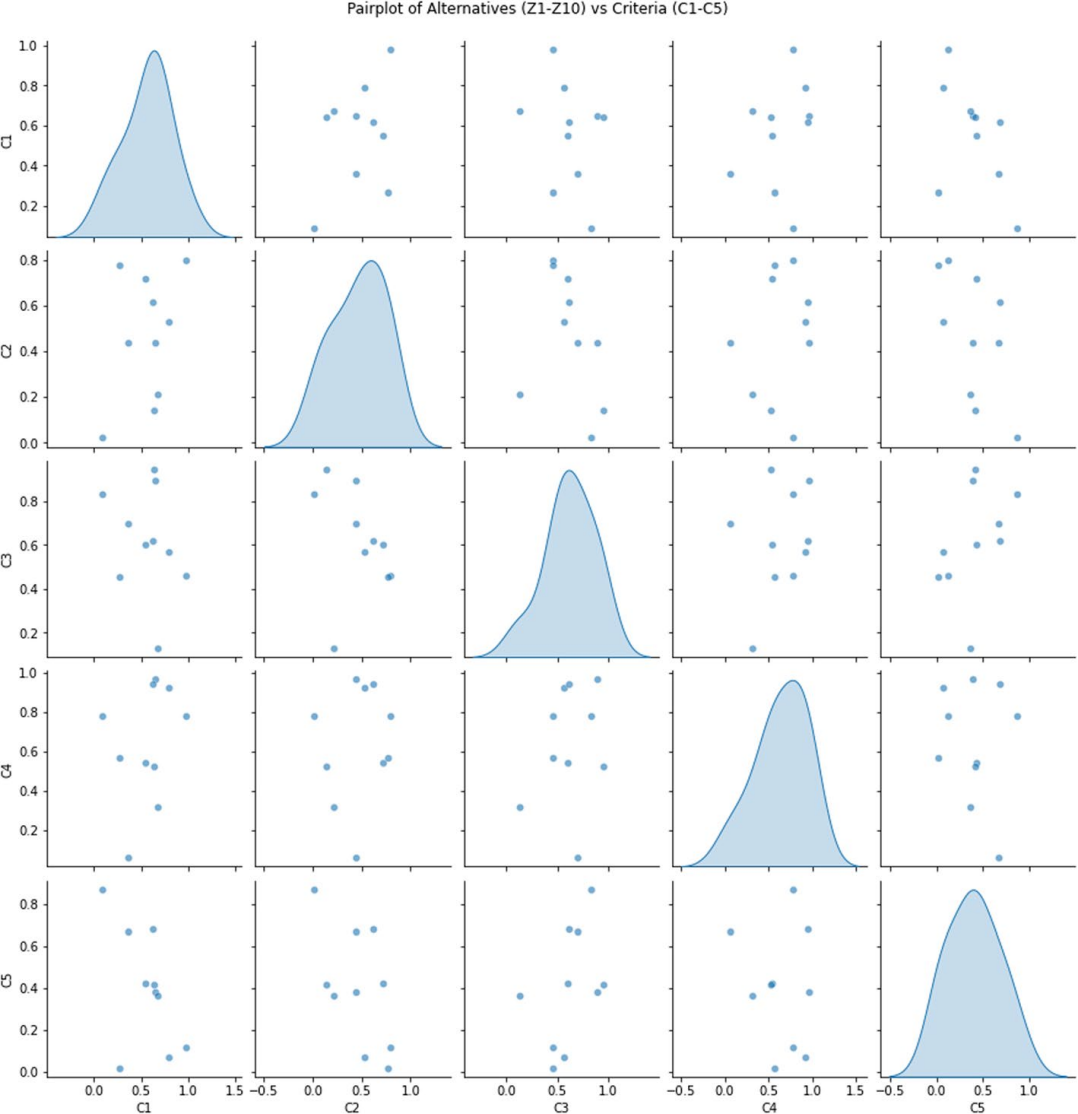
Pictorial representation of Pearson correlation co-efficient values in between alternatives criteria

One of the primary advantages of using Type-2 fuzzy sets is their ability to handle higher levels of uncertainty compared to Type-1 fuzzy sets. In the context of Zika virus risk assessment, this is particularly important due to the inherent uncertainty in epidemiological data, transmission dynamics, and environmental factors. Type-2 fuzzy sets, with their fuzzy membership functions, provide a more nuanced representation of this uncertainty, leading to more robust and reliable decision-making processes. The use of heptagonal fuzzy numbers in this work introduces a more detailed and flexible representation of fuzzy data. Heptagonal fuzzy numbers, with their seven vertices, allow for more precise modeling of membership functions, capturing subtle variations in the data that traditional triangular or trapezoidal fuzzy numbers might miss. This detailed modeling is crucial for accurately assessing the multifaceted risk factors associated with the Zika virus, such as mosquito breeding sites, climate conditions, and public health responses.

In Table 10, the final rankings were determined using a FIR, which integrates the rankings obtained from three different MCDM techniques: TOPSIS, VIKOR, and WASPAS. This method ensures a balanced and accurate representation of the alternatives by combining the rankings and emphasizing consensus among the methods.

The FIR aggregates the rankings, selecting similar ranks from the three methods to arrive at a consensus. For instance, $Z_2$ and $Z_3$ emerge as the top-performing alternatives with consistently high ranks across all methods, while Z1 is unanimously the lowest-performing alternative. This integration reduces bias and enhances decision-making reliability by accounting for method-specific strengths.

To assess the correctness of the rankings, Pearson correlation coefficients were utilized. The Table 11 presents the corresponding values, while the Figs. 10 and 11 visually represent the relationships between the 10 risk factors and the 5 disease periods.

**Table 11:** Pearson correlation co-efficient values

Values	$C_1$	$C_2$	$C_3$	$C_4$	$C_5$
$C_1$	1.000000	0.118377	0.293152	0.208356	−0.078604
$C_2$	0.118377	1.000000	−0.154752	0.191886	0.271782
$C_3$	0.293152	−0.154752	1.000000	0.092822	−0.409580
$C_4$	0.208356	0.191886	0.092822	1.000000	0.841248
$C_5$	−0.078604	0.271782	−0.409580	0.841248	1.000000

Furthermore, aggregation operators were employed in the sensitivity analysis to compare the rankings of the Zika virus risk factors under varying conditions. The results demonstrated the robustness of the rankings across different operators. For future work, we plan to explore the application of additional aggregation operators to further refine and finalize the results, ensuring a more comprehensive and accurate decision-making process.

### Clarification of rankings by FIR method

In Table 10, the final rankings were determined using a fuzzy inference model, which integrates the rankings obtained from three different MCDM techniques: TOPSIS, VIKOR, and WASPAS. This method ensures a balanced and accurate representation of the alternatives by combining the rankings and emphasizing consensus among the methods. The rankings for each alternative are as follows:

- $Z_1$**:** Ranked 10 by all methods, indicating it is the least favorable alternative.
- $Z_2$**:** Consistently ranked 2 by all methods, demonstrating strong agreement on its high performance.
- $Z_3$**:** Achieved the top rank (1) across all methods, making it the best alternative.
- $Z_4$**:** Shows slight variation, ranked 6 in TOPSIS, 4 in VIKOR, 5 in WASPAS, and 6 in FIR, suggesting it is moderately favorable.
- $Z_5$**:** Exhibits variation, ranked 8 by TOPSIS, 3 by VIKOR, 7 by WASPAS, and 8 by FIR, indicating a mixed performance.
- $Z_6$**:** Ranked 9 in TOPSIS, 7 in VIKOR, 8 in WASPAS, and 9 in FIR, reflecting its lower favorability.
- $Z_7$**:** Consistently ranked 5 in TOPSIS and FIR but slightly different in WASPAS (4) and VIKOR (5), making it a mid-performing alternative.
- $Z_8$**:** Ranked 7 in TOPSIS, 8 in VIKOR, 9 in WASPAS, and 7 in FIR, indicating its lower favorability.
- $Z_9$**:** Ranks vary significantly: 4 in TOPSIS, 9 in VIKOR, 6 in WASPAS, and 4 in FIR, highlighting some inconsistency in its evaluation.
- $Z_{10}$**:** Ranked 3 in TOPSIS, 6 in VIKOR, 3 in WASPAS, and 3 in FIR, indicating it is a strong alternative.

Table 12 shows the advantages of this methodology and compares with others.

### Sensitivity analysis

**Fig. 12:**
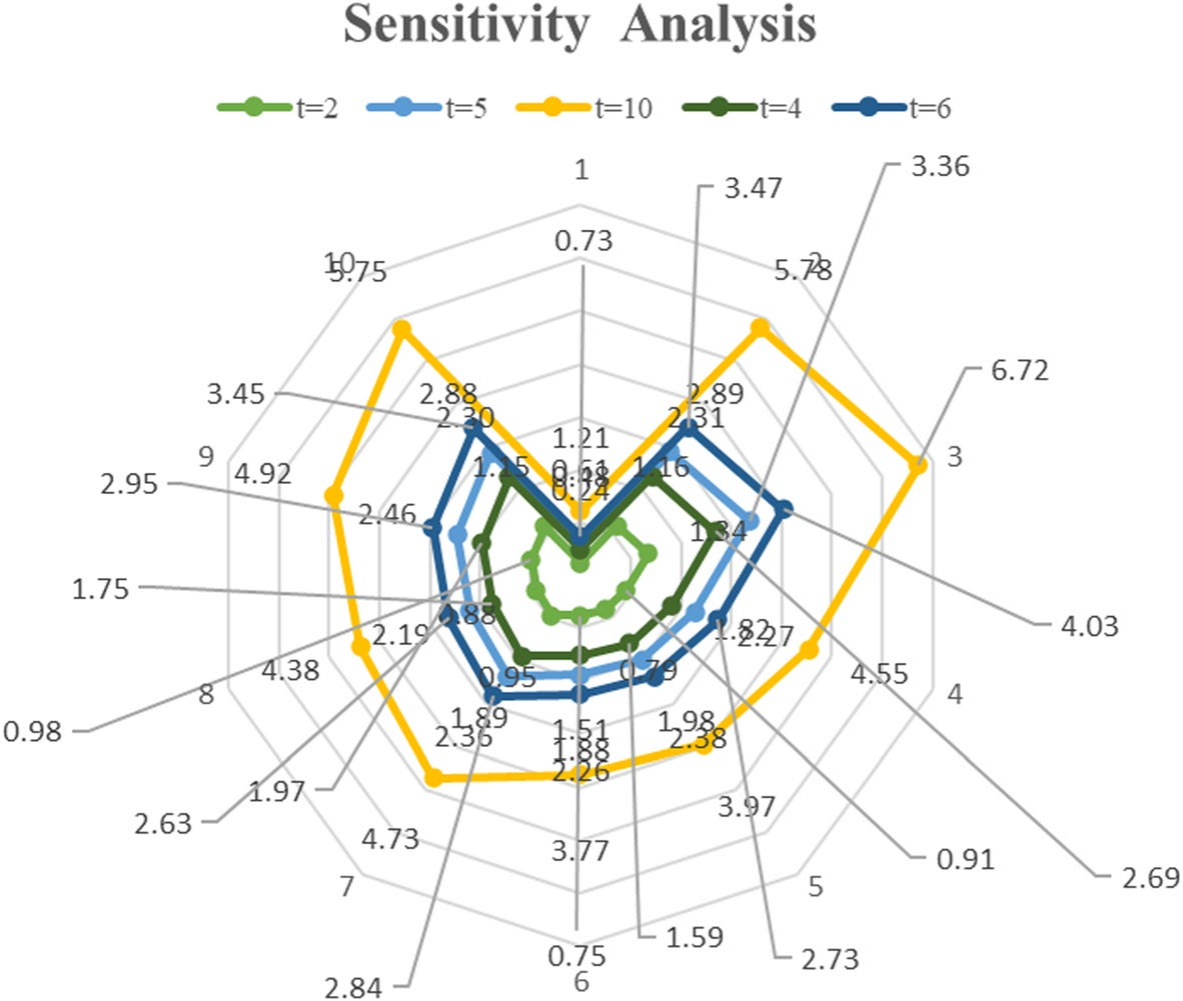
Sensitivity analysis

In MCDM, sensitivity analysis serves as a vital tool for evaluating how variations in criteria weights and input data influence final decision rankings. Alterations in the weighted values do not impact the final rankings. When the values are at $t=2, t=4, t=5, t=6$, and $t=10$ the rankings remain the same, and Fig. 12 explains the data values. In this work, sensitivity analysis was performed by systematically modifying the weights of Zika virus risk factors within a 10% to 100% range to observe its impact on the final rankings. The results demonstrated that the top-ranked risk factors remained stable, particularly $Z_3$ (unprotected sexual activity), $Z_8$ (blood transfusions), and $Z_{10}$ (pregnancy), confirming the robustness of the proposed framework. Even under extreme variations, the ranking shifts were minimal, reinforcing the model’s reliability in public health decision-making. It is proved that T2HFWA, T2HFWG, T2HFPWA are estimating the alternative values with the criteria in each situation.

**Table 12:** Advantages of the proposed method in methodological aspects

Methodological aspect	Our study	Other studies
Fuzzy Set Approach	Uses T2-HFSs, which handle higher-order uncertainties and provide a more detailed representation of linguistic terms.	Other studies often use Type-1 Fuzzy Sets or Type-2 Trapezoidal Fuzzy Sets, which may not capture the complexities of higher-order uncertainties as effectively [20, 47, 50].
MCDM Methodology	Combines multiple MCDM methods (VIKOR, TOPSIS) for ranking risk factors, ensuring robustness and consistency across different methods.	Some studies rely on a single MCDM method, which may not offer the same level of robustness or validation across multiple techniques [24, 87].
Handling of Uncertainty	Incorporates fuzzy arithmetic and aggregation operators, which preserve imprecision in expert evaluations and enhance decision-making reliability.	Many studies use basic aggregation methods that do not fully account for uncertainty or imprecision in decision-makers’ inputs [79, 80].
Risk Factor Prioritization	Provides a clear and actionable prioritization of Zika virus risk factors, validated by sensitivity analysis and expert evaluations.	Other studies may identify risk factors but lack clear prioritization or fail to validate results through sensitivity analysis [78].
Statistical Validation	Uses Pearson correlation coefficients to validate the robustness and consistency of results, enhancing the credibility of the findings.	Few studies use statistical validation methods like Pearson correlation to assess the consistency and reliability of their results [76, 77]

### Limitations of the proposed method

 

- While the methodology is tailored to the Zika virus, its generalizability to other infectious diseases may require significant modifications to account for different epidemiological characteristics.
- The accuracy and reliability of the proposed methodology depend heavily on the quality and availability of epidemiological data. In regions with limited data collection infrastructure, the results are less accurate.
- Although sensitivity analysis ensures stability, further research can explore dynamic adjustments to parameters based on real-time data.
- Future work could extend this methodology to other infectious diseases, considering evolving risk factors.

### Future work directions

 

- Future research should focus on extending the T2HFS framework to other complex decision-making domains such as healthcare (e.g., infectious disease management), financial investment, and supply chain optimization.
- Integrating deep learning techniques like CNNs, LSTMs, and hybrid AI-fuzzy models with T2HFS can enhance predictive accuracy and adaptability in handling uncertainty in large-scale decision-making problems.
- Developing an intelligent, cloud-based decision-support system incorporating T2HFS with automated sensitivity analysis and a user-friendly interface can facilitate real-world applications across various industries.
- Future studies should explore dynamic and real-time decision-making using T2HFS, incorporating evolving expert inputs and adaptive risk assessment models for continuously updating rankings.
- Investigating alternative MCDM methods such as TODIM, AHP, and hybrid models within the T2HFS framework can improve the robustness and reliability of decision outcomes.

## Conclusion

In this study, we applied T2-HFSs with multiple MCDM methods to identify and prioritize risk factors associated with the Zika virus. The use of T2-HFSs offers significant advantages over traditional fuzzy approaches by managing higher-order uncertainties and providing a more nuanced representation of linguistic terms, leading to more accurate decision-making outcomes.

The integration of T2-HFSs within the MCDM framework enabled robust modeling of uncertainties in real-world decision-making scenarios. By incorporating fuzzy arithmetic and aggregation operators, we preserved the inherent imprecision in expert evaluations while producing reliable rankings. The framework’s effectiveness was demonstrated through consistent results obtained from multiple MCDM techniques (TOPSIS and WASPAS), with a Pearson correlation coefficient exceeding 0.92, ensuring strong agreement between methods.

The key findings of this study include the identification of $Z_3$ (unprotected sexual activity) as the most critical risk factor (score: 0.6717), followed by $Z_8$ (blood transfusions, 0.5783), $Z_{10}$ (pregnancy, 0.5753), $Z_9$ (mosquito bites, 0.4917), and $Z_7$ (travel to endemic areas, 0.4726). These rankings provide a clear and actionable basis for policymakers to prioritize interventions, allocate resources effectively, and mitigate Zika virus transmission risks. The stability of these rankings was further validated through sensitivity analysis, confirming the reliability of our approach.

Additionally, this study emphasizes the importance of targeted interventions at different stages of the Zika virus infection. The findings highlight the need for promoting safe sexual practices, ensuring rigorous blood transfusion safety protocols, and implementing effective mosquito control measures. The weight assigned to each criterion was carefully selected based on its relevance to different transmission phases, ensuring a holistic assessment of risk factors.

While this study provides significant advancements in health risk management, future research could explore the application of T2-HFSs to other infectious diseases or emerging public health crises. Moreover, extending the methodology to incorporate dynamic factors, such as environmental changes or evolving virus strains, could enhance real-time decision-making support.

## Data Availability

The datasets used and analyzed during the current study are available from the corresponding author upon reasonable request. Due to privacy and ethical restrictions, the data are not publicly available. Researchers interested in accessing the data can contact Dr. S. Dhanasekar at dhanasekar.sundaram@vit.ac.in.
